# Causal effects of specific gut microbiota on bone mineral density: a two-sample Mendelian randomization study

**DOI:** 10.3389/fendo.2023.1178831

**Published:** 2023-08-14

**Authors:** Shuai Chen, Guowei Zhou, Huawei Han, Jie Jin, Zhiwei Li

**Affiliations:** ^1^ Department of Orthopaedics, The Second Affiliated Hospital of Nanjing University of Chinese Medicine, Nanjing, China; ^2^ Department of General Surgery, Jiangsu Province Hospital of Chinese Medicine, Affiliated Hospital of Nanjing University of Chinese Medicine, Nanjing, China

**Keywords:** osteoporosis, gut microbiota, bone mineral density, Mendelian randomization, causality

## Abstract

**Background:**

Recent studies have reported that the gut microbiota is essential for preventing and delaying the progression of osteoporosis. Nonetheless, the causal relationship between the gut microbiota and the risk of osteoporosis has not been fully revealed.

**Methods:**

A two-sample Mendelian randomization (MR) analysis based on a large-scale genome-wide association study (GWAS) was conducted to investigate the causal relationship between the gut microbiota and bone mineral density (BMD). Instrumental variables for 211 gut microbiota taxa were obtained from the available GWAS meta-analysis (n = 18,340) conducted by the MiBioGen consortium. The summary-level data for BMD were from the Genetic Factors for Osteoporosis (GEFOS) Consortium, which involved a total of 32,735 individuals of European ancestry. The inverse variance-weighted (IVW) method was performed as a primary analysis to estimate the causal effect, and the robustness of the results was tested *via* sensitivity analyses by using multiple methods. Finally, a reverse MR analysis was applied to evaluate reverse causality.

**Results:**

According to the IVW method, we found that nine, six, and eight genetically predicted gut microbiota were associated with lumbar spine (LS) BMD, forearm (FA) BMD, and femoral neck (FN) BMD, respectively. Among them, the higher genetically predicted *Genus Prevotella9* level was correlated with increased LS-BMD [β = 0.125, 95% confidence interval (CI): 0.050–0.200, *P* = 0.001] and FA-BMD (β = 0.129, 95% CI: 0.007–0.251, *P* = 0.039). The higher level of genetically predicted *Family Prevotellaceae* was associated with increased FA-BMD (β = 0.154, 95% CI: 0.020–0.288, *P* = 0.025) and FN-BMD (β = 0.080, 95% CI: 0.015–0.145, *P* = 0.016). Consistent directional effects for all analyses were observed in both the MR-Egger and weighted median methods. Subsequently, sensitivity analyses revealed no heterogeneity, directional pleiotropy, or outliers for the causal effect of specific gut microbiota on BMD (*P* > 0.05). In reverse MR analysis, there was no evidence of reverse causality between LS-BMD, FA-BMD, and FN-BMD and gut microbiota (*P* > 0.05).

**Conclusion:**

Genetic evidence suggested a causal relationship between the gut microbiota and BMD and identified specific bacterial taxa that regulate bone mass variation. Further exploration of the potential microbiota-related mechanisms of bone metabolism might provide new approaches for the prevention and treatment of osteoporosis.

## 1 Introduction

Osteoporosis is a common metabolic osteopathy with characteristics of decreased bone mineral density (BMD), which can cause bone fragility and an increased risk of fracture (1, 2). As the population ages, approximately 200 million individuals worldwide suffer from osteoporosis. By 2050, the prevalence of osteoporosis in men and women is expected to increase by 3.1- and 2.4-fold, respectively (3, 4). Osteoporotic fracture is a serious clinical complication of osteoporosis, which often occurs in the vertebra, hip, distal forearm (FA), and pelvis. Due to its high mortality and disability rates, it has a considerable detrimental influence on patients’ quality of life (5). Moreover, the annual cost of treating osteoporosis and its related bone fractures is estimated to increase from $10 billion in 2010 to $17 billion in 2030 (6, 7). Therefore, the prevention and management of osteoporosis has been globally recognized as an important public health issue that needs to be solved urgently.

The gut microbiota is a massive complex community of microbial species inhabiting the human gastrointestinal tract, which is closely related to nutrition, immunity, inflammation, and various diseases (8, 9). On the one hand, growing evidence suggests that the gut microbiota could regulate the T regulatory cell (Treg)/T helper cell (Th)17 cell balance or relevant cytokines through the immune system, affecting the intestinal and systemic immune states, thereby establishing a dynamic balance between osteoblasts (OBs) and osteoclasts (OCs) (10). On the other hand, microbial metabolites such as saturated fatty acids (SFAs), secondary bile acid (SBA), and indole derivatives participate in the reconstruction of bone resorption, metabolism, and fracture healing by providing energy to gut epithelium cells and promoting calcium and phosphorus absorption (11). According to a study by Xu et al. (12), the gut microbiota compositions of patients with osteoporosis were significantly different from those of healthy controls, especially the enriched *Dialister* and *Faecalibacterium* genera. In addition, Ma et al. (13) also showed that transplanting intestinal flora from young rats into old rats can improve intestinal homeostasis at the phylum and family levels and increase trabecular number, trabecular thickness, and bone volume fraction, demonstrating that the gut microbiota could directly affect bone metabolism (13). However, these studies are mainly based on observational and cross-sectional analyses, and it is still not clear whether there is a causal relationship between the gut microbiome and osteoporosis.

Mendelian randomization (MR) is a groundbreaking analytical method that provides an unbiased estimation of the causal link between phenotypes (14). Compared with traditional epidemiological studies, the MR study uses genetic variation as instrumental variables (IVs) to avoid the influence of traditional confounding factors, which provides robust evidence on the mechanisms of the pathogenesis of disease and the efficacy of treatments (15, 16). Therefore, we conduct a bidirectional two-sample MR analysis to investigate the causality of specific gut microbiota and BMD and to identify specific causal bacterial taxa based on genome-wide association study (GWAS) data.

## 2 Materials and methods

### 2.1 Study design

Summary-level data from published GWASs and the Genetic Factors for Osteoporosis (GEFOS) Consortium website were used in our study. Based on the bidirectional two-sample MR analysis, we evaluated the causal relationship between gut microbial genera and BMD. Our first step was to determine whether the gut microbiome contributes to the prevention or promotion of BMD by selecting the gut microbiome as the exposure and BMD as the outcome. Furthermore, we examined changes in the gut microbiota following the change in BMD. In the MR analysis, the following three assumptions should be met: 1) The instruments of genetic variations should be robustly associated with the gut microbiota; 2) The genetic variations should not be associated with any confounders of the gut microbiota and BMD nor with osteoporosis; 3) The genetic variations should affect BMD solely through the gut microbiota, not *via* other pathways (17).

### 2.2 Sources of genome-wide association studies

The summary data of the gut microbiota were derived from a large-scale multi-ethnic GWAS meta-analysis, which included 18,340 European individuals and individuals from 24 cohorts (MiBioGen Consortium). Three different variable regions of the 16S rRNA gene were targeted in order to profile the microbial composition. Only the taxa found in >10% of the samples were included in the quantitative microbiome trait loci (mbQTL) mapping study for each cohort. Furthermore, after adjustment for age, sex, technical covariates, and genetic principal components, Spearman’s correlation analysis was conducted to identify genetic loci that affected the covariate-adjusted abundance of bacterial taxa (18).

The GEFOS Consortium is a large international collaboration made up of various research organizations. BMD is a highly heritable trait and is an essential index of bone strength, in which genetic determinants may explain nearly 83%, 73%, and 75% of the variance in BMD at the sites of the lumbar spine (LS), forearm (FA), and femoral neck (FN), respectively (19). Therefore, we collected the published data on BMD from the GEFOS, which identified novel loci for BMD (g/cm^2^) derived from Dual-energy x-ray absorptiometry (DXA) at the FN (n = 32,735), LS (n = 28,498), and FA (n = 8,143) of 53,236 European participants (20). Detailed information on the demographic characteristics of selected summary-level GWASs applied in the MR study was shown in **Table 1**.

**Table 1:** Detailed characteristics of GWAS associated with exposures and outcomes in the study.

Traits	Consortium	Year	Population	Sample size	PMID
Exposure					
Gut microbiota	MiBioGen Consortium	2021	European	18,340	33462485
Outcome					
Lumbar spine BMD	Genetic Factors for Osteoporosis Consortium	2015	European	28,498	26367794
Forearm BMD	Genetic Factors for Osteoporosis Consortium	2015	European	8,143	26367794
Femoral neck BMD	Genetic Factors for Osteoporosis Consortium	2015	European	32,735	26367794

GWAS, genome-wide association study; PMID, PubMed unique identifier.

### 2.3 Selection of genetic instrumental variables

1) The IVs selected for analysis are highly related to the corresponding exposures [We chose significant single nucleotide polymorphisms (SNPs) based on a loose cutoff of *P* < 1 × 10^-5^ to ensure sufficient IVs for screening]. 2) The IVs are mutually independent and avoid the offset caused by linkage disequilibrium (LD) between the SNPs (r^2^ < 0.001, LD distance >10,000 kb). 3) We eliminated IVs with an F-statistic <10 to minimize potential weak instrument bias F = R^2^ (n-k-1)/k (1-R^2^) (n is the sample size, k is the number of included IVs, and R^2^ is the exposure variance explained by the selected SNPs).

### 2.4 Statistical analysis

The inverse variance-weighted (IVW) method was employed as the main analysis to obtain an unbiased estimate of the causal association between the gut microbiota and BMD. Furthermore, the weighted median, MR-Egger, simple mode, and weighted mode methods were applied as additional methods to estimate causal effects under different conditions. The weighted median could combine data from multiple genetic variants into a single causal estimate, providing a consistent estimate when at least 50% of weights are from valid IVs (21). The MR-Egger method, which allows all SNPs with horizontal pleiotropic effects to be unbalanced or directed, was used to estimate the causal effect of exposure on the outcome (22). The intercept of MR-Egger regression was calculated to assess horizontal pleiotropy, and *P* > 0.05 indicated that the possibility of a pleiotropy effect in causal analysis is weak. Cochran’s Q test was derived from IVW estimation and used to detect heterogeneity among IVs. In addition, we applied the Mendelian randomization pleiotropy residual sum and outlier (MR-PRESSO) method to determine horizontal pleiotropy and correct potential outliers (23). The leave-one-out method was used for the sensitivity analysis, which sequentially removed one of the SNPs and used the remaining SNPs as IVs for two-sample MR analysis to judge the degree of influence of the causal association effect by a single SNP. Finally, we also performed a reverse MR analysis to determine whether there was a reverse direction causal relationship. The “TwoSampleMR” package and the “MRPRESSO” package in R software (version 4.1.3) were used for all MR analyses.

## 3 Results

### 3.1 The selection of instrumental variables

To begin with, 14,587 (locus-wide significance level, *P* < 1 × 10^-5^) and 456 (genome-wide statistical significance threshold, *P* < 5 × 10^-8^) SNPs associated with the gut microbiota were identified as IVs from the MiBioGen Consortium. After a series of quality control steps, 2,929 (*P* < 1 × 10^-5^) and 18 (*P* < 5 × 10^-8^) SNPs were finally included in the analysis. In addition, as presented in **Supplementary Table S1**, the F-statistics of all IVs were >10, indicating no evidence of a weak instrument bias. The results of the associations between 211 bacterial traits and LS-BMD, FA-BMD, and FN-BMD were presented in **Supplementary Tables S2**–**S4**, respectively.

### 3.2 Causal effects of the gut microbiota on bone mineral density (locus-wide significance, *P* < 1 × 10^-5^)

#### 3.2.1 Causal effect of the gut microbiota on lumbar spine bone mineral density

**Figure 1:**
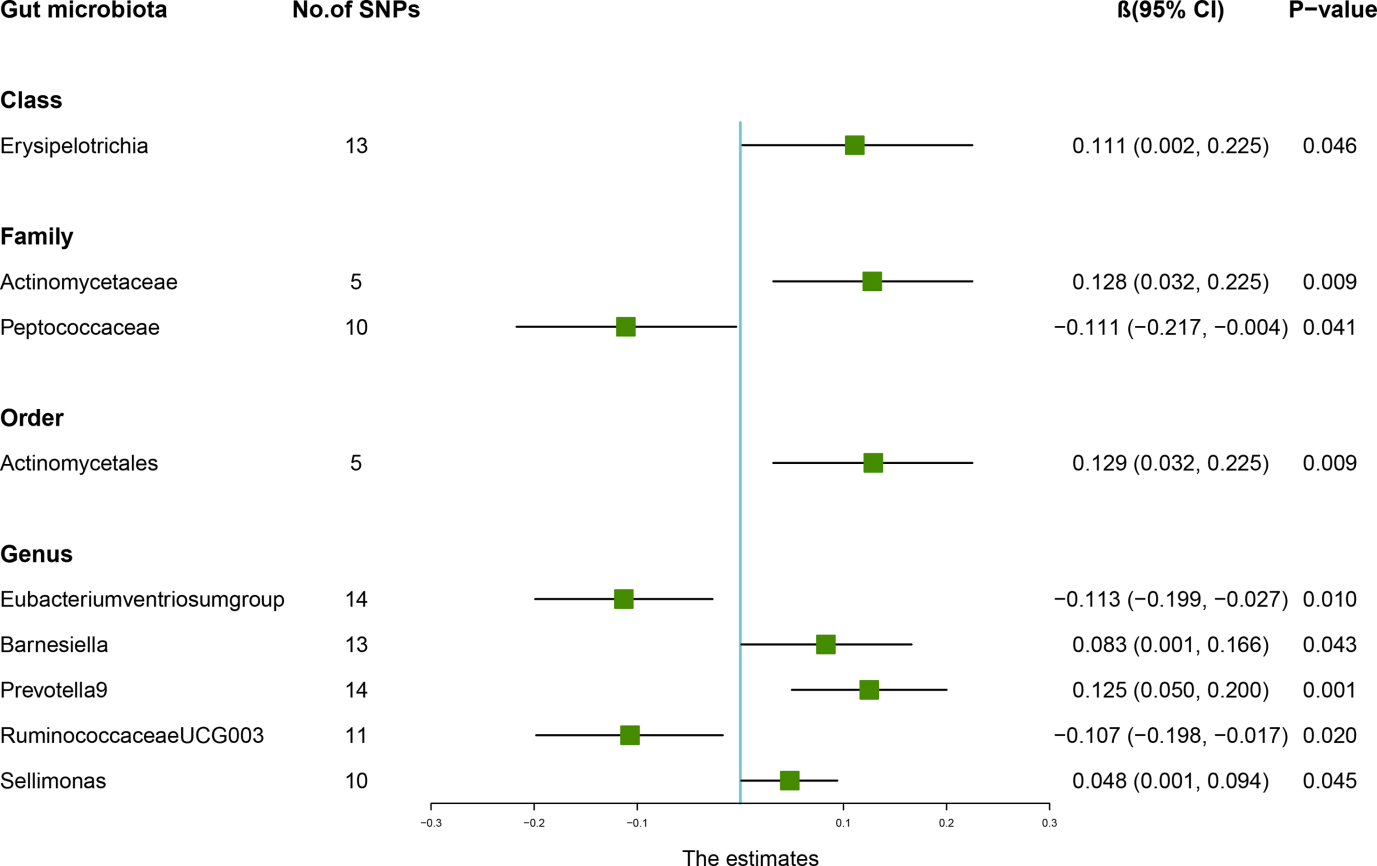
Associations between the gut microbiota and lumbar spine bone mineral density (BMD). The forest plot contains the effects, 95% CI, and *P*-values of all of the examined associations in analyses. Effect = the combined effect of the exposure on the BMD; *P*-value = *P*-value of the estimate.

According to the results of the IVW method, genetically predicted *Class Erysipelotrichia* [β = 0.111, 95% confidence interval (CI): 0.002–0.225, *P* = 0.046], *Family Actinomycetaceae* (β = 0.128, 95% CI: 0.032–0.225, *P* = 0.009), *Order Actinomycetales* (β = 0.129, 95% CI: 0.032–0.225, *P* = 0.009), *Genus Barnesiella* (β = 0.083, 95% CI: 0.001–0.166, *P* = 0.043), *Genus Prevotella9* (β = 0.125, 95% CI: 0.050–0.200, *P* = 0.001), and *Genus Sellimonas* (β = 0.048, 95% CI: 0.001–0.094, *P* = 0.045) were positively associated with LS-BMD (**Table 2**), and *Family Peptococcaceae* (β = -0.111, 95% CI: -0.217 to -0.004, *P* = 0.041), *Genus Eubacteriumventriosumgroup* (β = -0.113, 95% CI: -0.199 to -0.027, *P* = 0.010), and *Genus RuminococcaceaeUCG003* (β = -0.107, 95% CI: -0.198 to -0.017, *P* = 0.020) were negatively associated with LS-BMD (**Figure 1**). The MR estimates of the weighted median indicated that elevated levels of *Genus Prevotella9* (β = 0.127, 95% CI: 0.022–0.232, *P* = 0.018) and *Family Peptococcaceae* (β = -0.165, 95% CI: -0.277 to -0.053, *P* = 0.004) were related to increased and decreased LS-BMD, respectively. Based on the Cochran’s Q test, there was no evidence of heterogeneity for the effect of specific gut microbiota on LS-BMD (*P* > 0.05) (**Table 3**). All *P* values of the MR-Egger intercept tests were >0.05, which indicated no horizontal pleiotropy. Furthermore, we also did not discover any outliers through the MR-PRESSO global test (**Table 3**). Detailed scatter plots for each MR method analysis were shown in **Supplementary Figure S1**. Results from the leave-one-out analysis demonstrated that no SNP was an influential outlier (**Supplementary Figure S4**).

**Table 2:** MR estimates for the association between the gut microbiota and lumbar spine bone mineral density.

Group	Bacterial traits	Nsnp	Methods	SE	β (95% CI)	*P-*value
*Class*	*Erysipelotrichia*	13	MR-Egger	0.257	0.034 (-0.470, 0.538)	0.897
			Weighted median	0.072	0.165 (0.025, 0.306)	0.021*
			Inverse variance-weighted	0.057	0.111 (0.002, 0.225)	0.046*
			Simple mode	0.134	0.248 (-0.014, 0.511)	0.089
			Weighted mode	0.136	0.235 (-0.030, 0.501)	0.108
*Family*	*Actinomycetaceae*	5	MR-Egger	0.126	0.111 (-0.137, 0.358)	0.446
			Weighted median	0.063	0.071 (-0.053, 0.195)	0.263
			Inverse variance-weighted	0.049	0.128 (0.032, 0.225)	0.009*
			Simple mode	0.091	0.070 (-0.108, 0.247)	0.484
			Weighted mode	0.085	0.070 (-0.096, 0.236)	0.454
*Family*	*Peptococcaceae*	10	MR-Egger	0.140	-0.125 (-0.400, 0.150)	0.400
			Weighted median	0.057	-0.165 (-0.277, -0.053)	0.004*
			Inverse variance-weighted	0.054	-0.111 (-0.217, -0.004)	0.041*
			Simple mode	0.113	-0.206 (-0.428, 0.016)	0.102
			Weighted mode	0.084	-0.188 (-0.352, 0.024)	0.051
*Order*	*Actinomycetales*	5	MR-Egger	0.127	0.110 (-0.138, 0.358)	0.447
			Weighted median	0.064	0.071 (-0.055, 0.197)	0.269
			Inverse variance-weighted	0.049	0.129 (0.032, 0.225)	0.009*
			Simple mode	0.088	0.070 (-0.102, 0.241)	0.469
			Weighted mode	0.085	0.070 (-0.096, 0.236)	0.456
*Genus*	*Eubacteriumventriosumgroup*	14	MR-Egger	0.194	-0.105 (-0.486, 0.276)	0.598
			Weighted median	0.062	-0.111 (-0.232, 0.010)	0.071
			Inverse variance-weighted	0.044	-0.113 (-0.199, -0.027)	0.010*
			Simple mode	0.110	-0.143 (-0.358, 0.072)	0.215
			Weighted mode	0.114	-0.145 (-0.368, 0.078)	0.225
*Genus*	*Barnesiella*	13	MR-Egger	0.138	0.208 (-0.063, 0.478)	0.161
			Weighted median	0.055	0.085 (-0.023, 0.194)	0.124
			Inverse variance-weighted	0.043	0.083 (0.001, 0.166)	0.043*
			Simple mode	0.098	0.093 (-0.099, 0.285)	0.360
			Weighted mode	0.098	0.100 (-0.093, 0.292)	0.330
*Genus*	*Prevotella9*	14	MR-Egger	0.138	0.123 (-0.147, 0.393)	0.388
			Weighted median	0.054	0.127 (0.022, 0.232)	0.018*
			Inverse variance-weighted	0.038	0.125 (0.050, 0.200)	0.001*
			Simple mode	0.089	0.151 (-0.023, 0.326)	0.113
			Weighted mode	0.087	0.146 (-0.025, 0.317)	0.118
*Genus*	*RuminococcaceaeUCG003*	11	MR-Egger	0.149	-0.111 (-0.402, 0.181)	0.476
			Weighted median	0.061	-0.085 (-0.204, 0.033)	0.158
			Inverse variance-weighted	0.046	-0.107 (-0.198, -0.017)	0.020*
			Simple mode	0.105	-0.053 (-0.258, 0.152)	0.622
			Weighted mode	0.094	-0.059 (-0.243, 0.126)	0.546
*Genus*	*Sellimonas*	10	MR-Egger	0.132	0.117 (-0.141, 0.374)	0.402
			Weighted median	0.032	0.035 (-0.028, 0.098)	0.272
			Inverse variance-weighted	0.024	0.048 (0.001, 0.094)	0.045*
			Simple mode	0.058	0.046 (-0.069, 0.160)	0.454
			Weighted mode	0.056	0.042 (-0.067, 0.151)	0.470

SNP, single-nucleotide polymorphism; MR, Mendelian randomization; SE, standard error; 95% CI, 95% confidence interval.

*: P < 0.05.

**Table 3:** Sensitivity analysis of the MR analysis results of the gut microbiota and lumbar spine bone mineral density.

Group	Bacterial traits	Cochran’s Q statistic	Heterogeneity *P*-value	MR-Egger Intercept	Intercept *P*-value	MR-PRESSO Global test *P*-value
*Class*	*Erysipelotrichia*	15.628	0.209	0.006	0.724	0.235
*Family*	*Actinomycetaceae*	2.546	0.636	0.002	0.888	0.681
*Family*	*Peptococcaceae*	18.254	0.194	0.002	0.939	0.363
*Order*	*Actinomycetales*	2.551	0.636	0.002	0.886	0.690
*Genus*	*Eubacteriumventriosumgroup*	10.812	0.626	-0.000	0.981	0.658
*Genus*	*Barnesiella*	7.532	0.821	-0.011	0.362	0.826
*Genus*	*Prevotella9*	14.485	0.341	0.000	0.987	0.389
*Genus*	*RuminococcaceaeUCG003*	9.123	0.520	0.001	0.958	0.550
*Genus*	*Sellimonas*	8.032	0.430	-0.005	0.805	0.458

MR, Mendelian randomization.

#### 3.2.2 Causal effect of the gut microbiota on forearm bone mineral density

**Figure 2:**
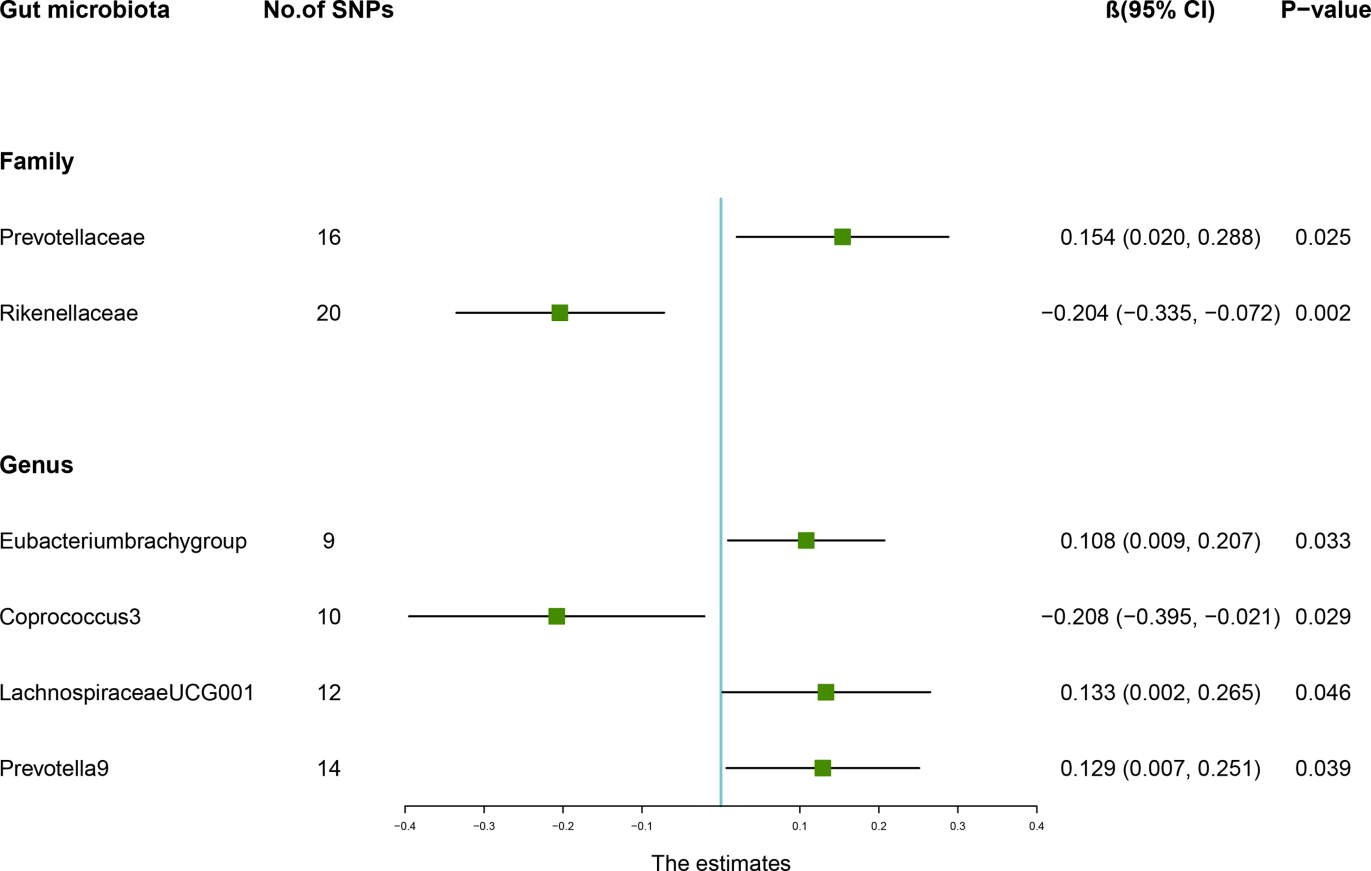
Associations between the gut microbiota and forearm bone mineral density (BMD). The forest plot contains the effects, 95% CI, and *P*-values of all of the examined associations in analyses. Effect = the combined effect of the exposure on the BMD; *P*-value = *P*-value of the estimate.

The estimates of the IVW test indicated that genetically predicted *Family Prevotellaceae* (β = 0.154, 95% CI: 0.020–0.288, *P* = 0.025), *Genus Eubacteriumbrachygroup* (β = 0.108, 95% CI: 0.009–0.207, *P* = 0.033), and *Genus LachnospiraceaeUCG001* (β = 0.133, 95% CI: 0.002–0.265, *P* = 0.046) were positively associated with FA-BMD (**Table 4**), and *Family Rikenellaceae* (β = -0.204, 95% CI: -0.335 to -0.072, *P* = 0.002), *Genus Coprococcus3* (β = -0.208, 95% CI: -0.395 to -0.021, *P* = 0.029), and *Genus Prevotella9* (β = 0.129, 95% CI: 0.007–0.251, *P* = 0.039) were negatively associated with FA-BMD (**Figure 2**). Futhermore, the estimates of weighted median suggested that genetically predicted *Family Prevotellaceae*, *Family Rikenellaceae*, and *Genus Eubacteriumbrachygroup* were causally related to FA-BMD (*P* < 0.05). The results of Cochran’s Q test indicated no significant heterogeneity (*P* > 0.05). The horizontal pleiotropy between specific gut microbiota and FA-BMD was evaluated by MR-Egger regression, showing no evidence of horizontal pleiotropy (**Table 5**). No outliers were discovered in the analysis of *Family Prevotellaceae* (*P* = 0.462), *Genus Eubacteriumbrachygroup* (*P* = 0.969), *Genus LachnospiraceaeUCG001* (*P* = 0.723), *Family Rikenellaceae* (*P* = 0.546), *Genus Coprococcus3* (*P* = 0.708), and *Genus Prevotella9* (*P* = 0.511) by MR-PRESSO (**Table 5**). Detailed scatter plots of the causal relationships between the gut microbiota and FA-BMD were presented in **Supplementary Figure S2**. Results from the leave-one-out analysis demonstrated that no SNP was an influential outlier (**Supplementary Figure S5**).

**Table 4:** MR estimates for the association between the gut microbiota and forearm bone mineral density.

Group	Bacterial traits	Nsnp	Methods	SE	β (95% CI)	*P-*value
*Family*	*Prevotellaceae*	16	MR-Egger	0.250	0.262 (-0.228, 0.751)	0.312
			Weighted median	0.096	0.194 (0.006, 0.383)	0.043*
			Inverse variance-weighted	0.068	0.154 (0.020, 0.288)	0.025*
			Simple mode	0.184	0.237 (-0.124, 0.598)	0.218
			Weighted mode	0.166	0.223 (-0.103, 0.549)	0.201
*Family*	*Rikenellaceae*	20	MR-Egger	0.205	-0.193 (-0.596, 0.209)	0.359
			Weighted median	0.089	-0.221 (-0.397, -0.046)	0.013*
			Inverse variance-weighted	0.067	-0.204 (-0.335, -0.072)	0.002*
			Simple mode	0.173	-0.233 (-0.573, 0.107)	0.195
			Weighted mode	0.194	-0.235 (-0.615, 0.145)	0.240
*Genus*	*Eubacteriumbrachygroup*	9	MR-Egger	0.216	0.166 (-0.258, 0.589)	0.468
			Weighted median	0.066	0.140 (0.011, 0.268)	0.033*
			Inverse variance-weighted	0.051	0.108 (0.009, 0.207)	0.033*
			Simple mode	0.114	0.165 (-0.058, 0.389)	0.185
			Weighted mode	0.117	0.162 (-0.068, 0.392)	0.205
*Genus*	*Coprococcus3*	10	MR-Egger	0.931	-0.860 (-2.685, 0.966)	0.383
			Weighted median	0.126	-0.187 (-0.434, 0.060)	0.138
			Inverse variance-weighted	0.095	-0.208 (-0.395, -0.021)	0.029*
			Simple mode	0.196	-0.207 (-0.590, 0.177)	0.319
			Weighted mode	0.204	-0.197 (-0.597, 0.202)	0.358
*Genus*	*LachnospiraceaeUCG001*	12	MR-Egger	0.304	0.252 (-0.343, 0.848)	0.426
			Weighted median	0.089	0.158 (-0.017, 0.333)	0.077
			Inverse variance-weighted	0.067	0.133 (0.002, 0.265)	0.046*
			Simple mode	0.160	0.225 (-0.090, 0.539)	0.189
			Weighted mode	0.157	0.222 (-0.086, 0.531)	0.186
*Genus*	*Prevotella9*	14	MR-Egger	0.211	0.006 (-0.420, 0.408)	0.978
			Weighted median	0.088	0.107 (-0.065, 0.279)	0.224
			Inverse variance-weighted	0.062	0.129 (0.007, 0.251)	0.039*
			Simple mode	0.128	0.032 (-0.220, 0.284)	0.807
			Weighted mode	0.126	0.071 (-0.177, 0.319)	0.585

SNP, single-nucleotide polymorphism; MR, Mendelian randomization; SE, standard error; 95% CI, 95% confidence interval.

*: P < 0.05.

**Table 5:** Sensitivity analysis of the MR analysis results of the gut microbiota and forearm bone mineral density.

Group	Bacterial traits	Cochran’s Q statistic	Heterogeneity *P*-value	MR-Egger Intercept	Intercept *P*-value	MR-PRESSO Global test *P*-value
*Family*	*Prevotellaceae*	14.959	0.454	-0.008	0.659	0.462
*Family*	*Rikenellaceae*	8.311	0.974	-0.000	0.977	0.969
*Genus*	*Eubacteriumbrachygroup*	5.405	0.714	-0.000	0.989	0.723
*Genus*	*Coprococcus3*	6.353	0.499	0.036	0.548	0.546
*Genus*	*LachnospiraceaeUCG001*	7.909	0.721	-0.010	0.714	0.708
*Genus*	*Prevotella9*	12.917	0.454	0.013	0.517	0.511

MR, Mendelian randomization.

#### 3.2.3 Causal effect of the gut microbiota on femoral neck bone mineral density

**Figure 3:**
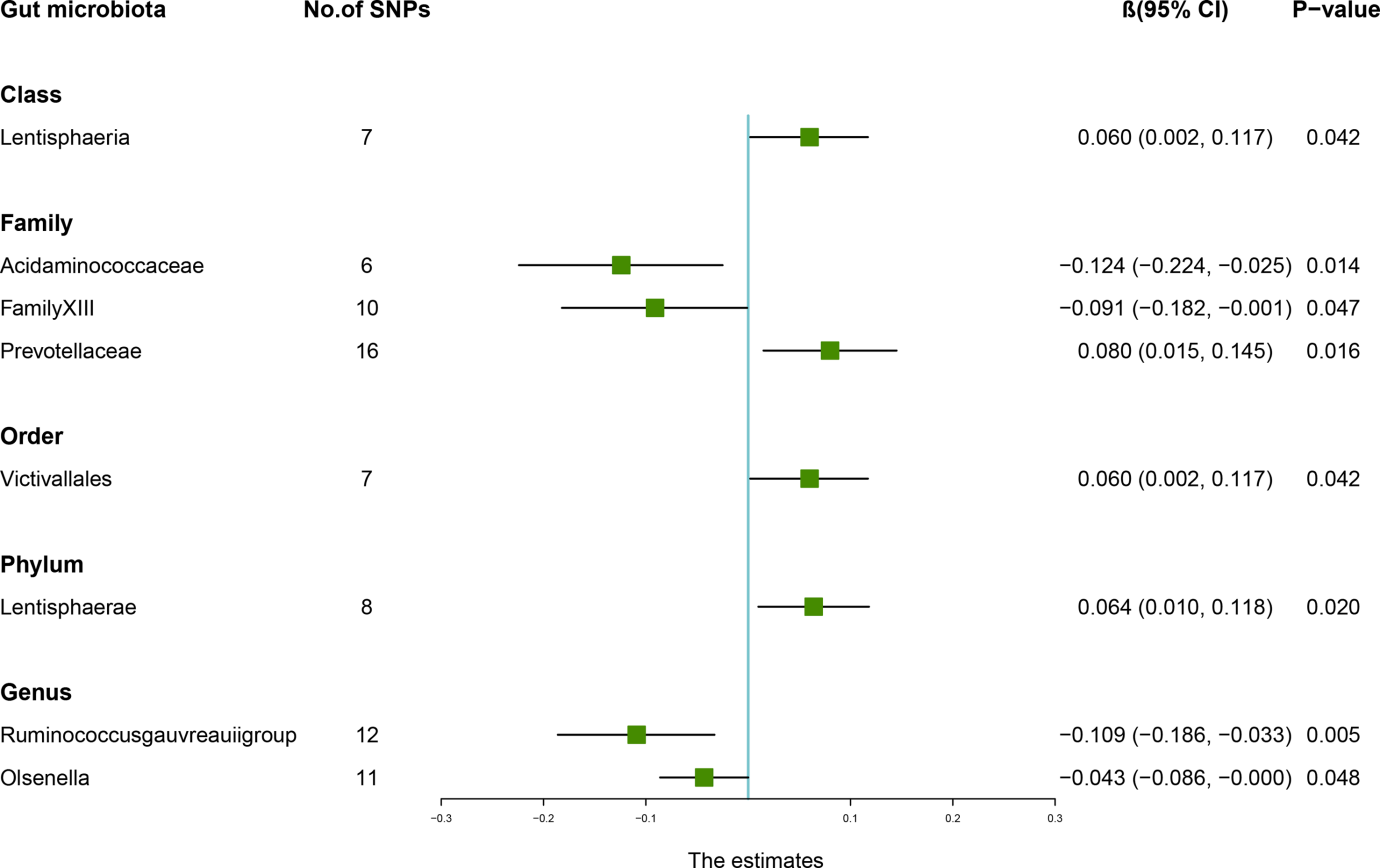
Associations between the gut microbiota and femoral neck bone mineral density (BMD). The forest plot contains the effects, 95% CI, and *P*-values of all of the examined associations in analyses. Effect = the combined effect of the exposure on the BMD; *P*-value = *P*-value of the estimate.

According to the IVW method, higher genetically predicted *Class Lentisphaeria* (β = 0.060, 95% CI: 0.002–0.117, *P* = 0.042), *Family Prevotellaceae* (β = 0.080, 95% CI: 0.015–0.145, *P* = 0.016), *Order Victivallales* (β = 0.060, 95% CI: 0.002–0.117, *P* = 0.042), and *Phylum Lentisphaerae* (β = 0.064, 95% CI: 0.010–0.118, *P* = 0.020) were linked to an increase in FN-BMD (**Table 6**). While *Family Acidaminococcaceae* (β = -0.124, 95% CI: -0.224 to -0.025, *P* = 0.014), *Family FamilyXIII* (β = -0.091, 95% CI: -0.182 to -0.001, *P* = 0.047), *Genus Ruminococcusgauvreauiigroup* (β = -0.109, 95% CI: -0.186 to -0.033, *P* = 0.005), and *Genus Olsenella* (β = -0.043, 95% CI: -0.086 to -0.000, *P* = 0.048) were associated with a decrease in FN-BMD (**Figure 3**). In addition, the Cochran’s Q test revealed no heterogeneity for the causal effect of specific gut microbiota on FN-BMD (*P* > 0.05). Based on the global test of MR-PRESSO and the intercept of MR-Egger, we excluded potential heterogeneity and horizontal pleiotropy in causal associations (**Table 7**). Detailed scatter plots of the causal correlations between the gut microbiota and FN-BMD were shown in **Supplementary Figure S3**. In addition, results from the leave-one-out analysis revealed that genetically predicted gut microbiota and FN-BMD remained substantially consistent after omitting one single SNP at a time (**Supplementary Figure S6**).

**Table 6:** MR estimates for the association between the gut microbiota and femoral neck bone mineral density.

Group	Bacterial traits	Nsnp	Methods	SE	β (95% CI)	*P-*value
*Class*	*Lentisphaeria*	7	MR-Egger	0.110	0.143 (-0.073, 0.359)	0.252
			Weighted median	0.037	0.039 (-0.033, 0.111)	0.289
			Inverse variance-weighted	0.029	0.060 (0.002, 0.117)	0.042*
			Simple mode	0.055	0.027 (-0.081, 0.135)	0.641
			Weighted mode	0.056	0.028 (-0.082, 0.138)	0.637
*Family*	*Acidaminococcaceae*	6	MR-Egger	0.164	-0.075 (-0.396, 0.246)	0.671
			Weighted median	0.063	-0.120 (-0.244, 0.003)	0.057
			Inverse variance-weighted	0.051	-0.124 (-0.224, -0.025)	0.014*
			Simple mode	0.097	-0.114 (-0.304, 0.076)	0.292
			Weighted mode	0.092	-0.111 (-0.291, 0.069)	0.280
*Family*	*FamilyXIII*	10	MR-Egger	0.166	-0.102 (-0.428, 0.224)	0.558
			Weighted median	0.060	-0.051 (-0.168, 0.066)	0.395
			Inverse variance-weighted	0.046	-0.091 (-0.182, -0.001)	0.047*
			Simple mode	0.100	-0.026 (-0.223, 0.170)	0.798
			Weighted mode	0.091	-0.024 (-0.202, 0.155)	0.803
*Family*	*Prevotellaceae*	16	MR-Egger	0.116	0.278 (0.050, 0.506)	0.031
			Weighted median	0.045	0.076 (-0.011, 0.164)	0.088
			Inverse variance-weighted	0.033	0.080 (0.015, 0.145)	0.016*
			Simple mode	0.076	0.080 (-0.070, 0.230)	0.311
			Weighted mode	0.067	0.095 (-0.036, 0.226)	0.175
*Order*	*Victivallales*	7	MR-Egger	0.110	0.143 (-0.073, 0.359)	0.252
			Weighted median	0.038	0.039 (-0.035, 0.114)	0.305
			Inverse variance-weighted	0.029	0.060 (0.002, 0.117)	0.042*
			Simple mode	0.056	0.027 (-0.084, 0.138)	0.649
			Weighted mode	0.055	0.028 (-0.079, 0.135)	0.628
*Phylum*	*Lentisphaerae*	8	MR-Egger	0.110	0.158 (-0.058, 0.375)	0.201
			Weighted median	0.037	0.049 (-0.023, 0.121)	0.184
			Inverse variance-weighted	0.028	0.064 (0.010, 0.118)	0.020*
			Simple mode	0.054	0.032 (-0.074, 0.138)	0.572
			Weighted mode	0.054	0.033 (-0.073, 0.139)	0.559
*Genus*	*Ruminococcusgauvreauiigroup*	12	MR-Egger	0.177	-0.111 (-0.457, 0.236)	0.546
			Weighted median	0.054	-0.051 (-0.158, 0.055)	0.345
			Inverse variance-weighted	0.039	-0.109 (-0.186, -0.033)	0.005*
			Simple mode	0.099	-0.028 (-0.223, 0.166)	0.782
			Weighted mode	0.089	-0.028 (-0.203, 0.147)	0.759
*Genus*	*Olsenella*	11	MR-Egger	0.086	-0.132 (-0.300, 0.037)	0.160
			Weighted median	0.030	-0.049 (-0.108, 0.010)	0.104
			Inverse variance-weighted	0.022	-0.043 (-0.086, -0.000)	0.048*
			Simple mode	0.049	-0.055 (-0.151, 0.041)	0.290
			Weighted mode	0.045	-0.055 (-0.142, 0.033)	0.250

SNP, single-nucleotide polymorphism; MR, Mendelian randomization; SE, standard error; 95% CI, 95% confidence interval.

*: P < 0.05.

**Table 7:** Sensitivity analysis of the MR analysis results of the gut microbiota and femoral neck bone mineral density.

Group	Bacterial traits	Cochran’s Q statistic	Heterogeneity *P*-value	MR-Egger Intercept	Intercept *P*-value	MR-PRESSO Global test *P*-value
*Class*	*Lentisphaeria*	2.339	0.886	-0.012	0.469	0.877
*Family*	*Acidaminococcaceae*	3.716	0.591	-0.005	0.767	0.641
*Family*	*FamilyXIII*	4.533	0.873	-0.002	0.867	0.884
*Family*	*Prevotellaceae*	12.301	0.656	-0.014	0.097	0.680
*Order*	*Victivallales*	2.339	0.886	-0.012	0.469	0.881
*Phylum*	*Lentisphaerae*	2.554	0.923	-0.014	0.411	0.916
*Genus*	*Ruminococcusgauvreauiigroup*	5.916	0.879	-0.001	0.918	0.876
*Genus*	*Olsenella*	4.835	0.902	0.012	0.315	0.914

MR, Mendelian randomization.

#### 3.2.4 Causal effects of the gut microbiota on BMD (genome-wide statistical significance, *P* < 5 × 10^-8^)

When MR analysis was conducted with gut microbiome as a whole, IVW results indicated that higher genetically predicted gut microbiome was positively linked with LS-BMD (β = 0.087, 95% CI: 0.018–0.156, *P* = 0.013), FA-BMD (β = 0.100, 95% CI: 0.002–0.199, *P* = 0.045), and FN-BMD (β = 0.048, 95% CI: 0.001–0.095, *P* = 0.047) (**Table 8**). There was a tendency toward a protective impact of increasing genetically predicted total gut microbiome on FN-BMD in the MR-Egger and weighted median methods. Additionally, the results of Cochran’s Q statistics indicated no significant heterogeneity (*P* > 0.05), and MR-Egger regression demonstrated no horizontal pleiotropy between the total gut microbiome and BMD. Finally, we also did not discover any outliers through the MR-PRESSO global test (**Table 9**).

**Table 8:** MR estimates for the association between the total gut microbiota and LS-BMD, FA-BMD, and FN-BMD.

Traits (outcome)	Bacterial traits (exposure)	Nsnp	Methods	SE	β (95% CI)	*P-*value
LS-BMD	*Total*	12	MR-Egger	0.117	-0.048 (-0.276, 0.181)	0.692
			Weighted median	0.039	0.033 (-0.043, 0.110)	0.395
			Inverse variance-weighted	0.035	0.087 (0.018, 0.156)	0.013*
			Simple mode	0.064	0.061 (-0.065, 0.188)	0.364
			Weighted mode	0.046	0.021 (-0.070, 0.111)	0.659
FA-BMD	*Total*	12	MR-Egger	0.168	-0.172 (-0.502, 0.158)	0.330
			Weighted median	0.071	0.030 (-0.109, 0.170)	0.671
			Inverse variance-weighted	0.050	0.100 (0.002, 0.199)	0.045*
			Simple mode	0.109	0.073 (-0.141, 0.288)	0.517
			Weighted mode	0.087	0.018 (-0.152, 0.189)	0.838
FN-BMD	*Total*	12	MR-Egger	0.082	0.009 (-0.152, 0.171)	0.912
			Weighted median	0.035	0.029 (-0.040, 0.097)	0.412
			Inverse variance-weighted	0.024	0.048 (0.001, 0.095)	0.047*
			Simple mode	0.050	0.030 (-0.068, 0.127)	0.561
			Weighted mode	0.046	0.028 (-0.061, 0.118)	0.548

SNP, single-nucleotide polymorphism; MR, Mendelian randomization; SE, standard error; 95% CI, 95% confidence interval; BMD, bone mineral density; LS, lumbar spine; FA, forearm; FN, femoral neck.

*: P < 0.05.

**Table 9:** Sensitivity analysis of the MR analysis results of the total gut microbiota and LS-BMD, FA-BMD, and FN-BMD.

Traits (outcome)	Bacterial traits (exposure)	Cochran’s Q statistic	Heterogeneity *P*-value	MR-Egger Intercept	Intercept *P*-value	MR-PRESSO Global test *P*-value
LS-BMD	*Total*	14.942	0.134	0.015	0.254	0.108
FA-BMD	*Total*	8.370	0.593	0.030	0.121	0.436
FN-BMD	*Total*	8.811	0.550	0.004	0.634	0.577

MR, Mendelian randomization; BMD, bone mineral density; LS, lumbar spine; FA, forearm; FN, femoral neck.

#### 3.2.5 Reverse Mendelian randomization analysis

The results of reverse MR analysis were shown in **Supplementary Table S6**. Considering cross-validation, we did not observe any reverse causal relationships between LS-BMD, FA-BMD, and FN-BMD and the gut microbiota (*P* > 0.05). In addition, sensitivity analyses revealed no heterogeneity, directional pleiotropy, or outliers for the causal effect of BMD on specific gut microbiota (*P* > 0.05).

## 4 Discussion

To our knowledge, this was the first comprehensive and in-depth investigation of causal associations between the gut microbiota and BMD based on publicly available GWAS data. According to the findings of our study, a total of 21 gut microbiota were potentially causally associated with BMD and the progression of osteoporosis. A higher genetically predicted gut microbiome was positively correlated with BMD at different skeletal sites (LS-BMD, FA-BMD, and FN-BMD). These findings might provide new ideas for osteoporosis management and treatment by targeting specific gut microbiota in the future.

The gut microbiota consists of trillions of bacteria in the gastrointestinal tract, which has functions such as improving intestinal permeability, attenuating the inflammatory response, and participating in immune regulation of the skeletal system (24). In recent years, the “gut–bone” axis has been receiving increasing attention in the field of bone health and orthopedic diseases, and multiple studies indicated that the composition of the gut microbiota regulates bone metabolism through multiple pathways (25). An experimental animal study demonstrated that *Erysipelotrichia*, *Enterobacteriales*, *Actinomycetales*, and *Ruminococcus* were significantly associated with serum biomarkers related to bone metabolism, such as serum bone Gla-protein (BGP), osteoprotegerin (OPG), and tartrate-resistant acid phosphatase (TRACP), which have beneficial effects on improving bone microarchitecture (26). In contrast, Huang et al. (27) observed that the relative abundance of *Ruminococcus* was higher in osteoporosis patients compared with that in healthy patients. Similarly, another animal study found that *Ruminococcus* in ovariectomized (OVX) rats was significantly increased compared with that in the sham group and was positively associated with bone loss (28). Based on the findings reviewed above, we speculated that the different effects of *Ruminococcus* on osteoporosis may be species- and strain-specific, which warrants further investigation.

In addition, Nogal et al. (29) reported a close correlation between acetate and *Barnesiella*, and that acetate was a health-promoting molecule with bone and gut protective effects that enhances immunity and inhibits intestinal inflammation and OC differentiation, thereby regulating bone metabolism. According to some literature, *LachnospiraceaeUCG001* belonged to the *Lachnospiraceae* family of bacteria, and it might play an anti-inflammatory role by controlling metabolite (such as short-chain fatty acids, SBA, indole derivatives, and polyamines) production (30). Moreover, we also found that some innovative gut bacteria, including *Lentisphaeria*, *Victivallales*, and *Lentisphaerae*, were associated with the levels of BMD. However, the exact mechanism of their effect on osteoporosis needs further study.

Our MR analysis also found that the abundance of *Prevotella9* and *Prevotellaceae* was associated with high BMD at different sites. Consistent directional effects for all analyses were observed in both MR-Egger and weighted median methods, which suggests that *Prevotella* might be a promising target for osteoporosis prevention. *Prevotella* was a commensal bacterium that widely exists in the human gut, oral, and reproductive tracts (31). In a study by Wang et al. (32), altered richness and a decreased proportion of *Prevotella* in the postmenopausal osteoporosis (PMO) group relative to the normal bone mass group and transplantation of *Prevotella* into OVX mice were effective at slowing bone loss. Furthermore, several mechanism studies have indicated that *Prevotella* reduces the intestinal permeability of estrogen-deficient mice by upregulating the expression of tight junction proteins including zonula occludens-1 (ZO-1) and occludin, which further prevents the release of inflammatory cytokines. Together, these effects inhibited osteoclastic bone resorption and prevented bone loss through the “gut–bone” axis (33).

Our study based on genetic prediction found that there were causal relationships between several gut microbial taxa and the decline of BMD, some of which have been confirmed in previous observational studies. Wei et al. (34) demonstrated that the absolute and relative abundances of *Clostridium_XLVa*, *Coprococcus*, and *Lactobacillus* were higher and the abundance of *Veillonella genus* was lower in osteoporosis patients compared with those in the controls. According to the results of another study, the abundance of *Eubacterium ventriosum group*, *Ruminococcus_1*, *Family_XIII*, and *Coprococcus* was negatively linked with the risk of rheumatoid arthritis, suggesting that these specific gut microbiota can increase matrix metallo-proteinase (MMP)-1, MMP-3, and MMP-13 expression, as well as OC activity to aggravate cartilage and bone damage (35). According to a case-control study, the level of *Family Rikenellaceae* was higher in the low BMD group and *Rikenellaceae* might have an adverse impact on bone resorption and bone density (36). Similarly, compared with the OVX group, the anti-osteoporosis treatment group increased the abundances of *Lactobacillus_reuteri*, *Muribaculaceae*, and *Clostridia* that were reported to increase bone mass and inhibited the relative abundance of *Rikenellaceae* (37).

Interestingly, when MR analysis was performed with the gut microbiome as a whole, the results of IVW indicated that a higher genetically predicted gut microbiome was positively linked with LS-BMD, FA-BMD, and FN-BMD. The gut microbiota was usually divided into beneficial conditional pathogen and pathogenic bacteria, and the balance between beneficial and pathogenic flora was critical for homeostasis and preventing bone metabolic diseases (38). He et al. (39) tested the gut microbiota of 106 postmenopausal women and found that the richness of the bacterial community was significantly lower in the osteoporosis group than that in the normal bone mass and osteopenia groups. Among them, *Enterobacter*, *Citrobacter*, *Pseudomonas*, and *Desulfovibrio* were enriched in postmenopausal osteopenia, while the osteoporosis group was more abundant in *Parabacteroides*, *Lactobacillus*, and *Actinomycetales*. Similarly, we found that the gut microbiota causally associated with BMD were mostly beneficial flora, suggesting that regulating the balance of the overall gut microbiota composition may be beneficial for improving bone health and reducing the risk of osteoporosis. In recent years, multiple studies have been published indicating that probiotics can be involved in the regulation of bone metabolism by mediating the production of immune inflammatory factors, improving intestinal barrier permeability, and regulating the metabolism of short-chain fatty acids (40–42). Li et al. (43) confirmed through animal experiments that supplementation of exogenous probiotics could reduce the serum concentration of tumor necrosis factor-á (TNF-á), receptor activator of NF-kB ligand (RANKL), interleukin 17A (IL-17A) in estrogen-deficient mice, attenuating systemic inflammatory responses, maintaining intestinal and whole body immune system balance, and slowing bone loss. In addition, a randomized double-blind controlled trial reported that multispecies probiotic supplementation for 6 months significantly reduced BMD loss caused by estrogen deficiency and improved bone turnover in postmenopausal women with osteopenia (44).

Our study had several strengths. First of all, we used MR analysis to infer that the correlation between the gut microbiome and BMD should be less susceptible to confounding and reverse causation than traditional observational analyses. Additionally, we analyzed the causal effect of each taxon on BMD from the genus to the phylum level, which provides guidance for the prevention and treatment of osteoporosis by targeting specific gut microbiota in clinical practice. However, the present study also had some limitations that should be noted. Firstly, due to the lack of demographic data in the original study (e.g., gender, age, and race), we were unable to perform further subgroup analyses to obtain more specific effect relationships. Secondly, the GWAS data on the gut microbiota used in this study are based on the population cohort from the largest macrogenome sequencing study to date. In the future, summary data of other gut microbiota need to be obtained for a more comprehensive assessment of the causal relationship between gut microbes and osteoporosis risk. Last but not least, this study was confined to individuals of European origin, and other populations require further MR studies, as causal relationships may vary from race to race.

## 5 Conclusion

In summary, we evaluated the causal relationship between the gut microbiota and BMD and identified potentially causal bacterial taxa that may be responsible for osteoporosis. However, further prospective cohort studies and mechanistic studies are needed to confirm these findings.

## Ethics approval and consent to participate

Ethical approval and informed consent for studies included in the analyses was provided in the original publications.

## Data availability statement

The original contributions presented in the study are included in the article/**Supplementary Material**. Further inquiries can be directed to the corresponding author.

## Author contributions

SC collected data. HH and JJ organized the study and performed the statistical analysis. GZ and ZL drafted the manuscript, to which all authors contributed, and approved the final version for publication. All authors contributed to the article and approved the submitted version.
