# Supplementary material for: Causal effects of specific gut microbiota on bone mineral density: a two-sample Mendelian randomization study
Source: Front Endocrinol (Lausanne). 2023 Aug 14;14:1178831. doi: 10.3389/fendo.2023.1178831 (PMC10461557; doi:10.3389/fendo.2023.1178831)

Supplementary Material

**Supplementary Figure S1.**

Scatter plots of causal estimates of exposure (Specific gut microbiota) on lumbar spine bone mineral density. The slope of each line corresponding to the estimated MR effect in different models, including the conventional IVW, Weighted median, MR-Egger, Simple mode, and Weighted mode. (A): *Class Erysipelotrichia*; (B): *Family Actinomycetaceae*; (C): *Family Peptococcaceae*; (D): *Order Actinomycetales*; (E): *Genus Barnesiella*; (F): *Genus Prevotella9*; (G): *Genus RuminococcaceaeUCG003*; (H): *Genus Sellimonas*; (I): *Genus Eubacteriumventriosumgroup* A
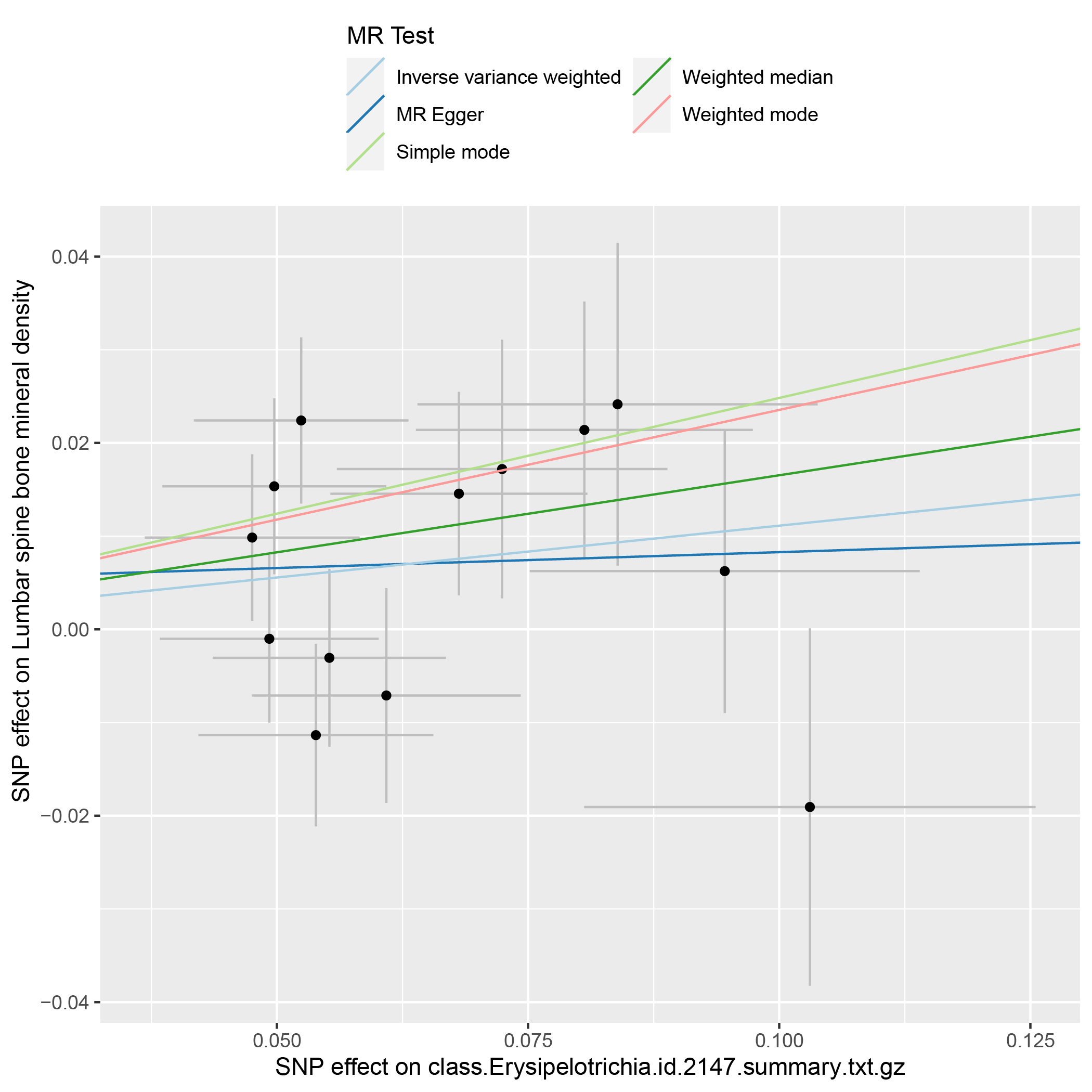
B
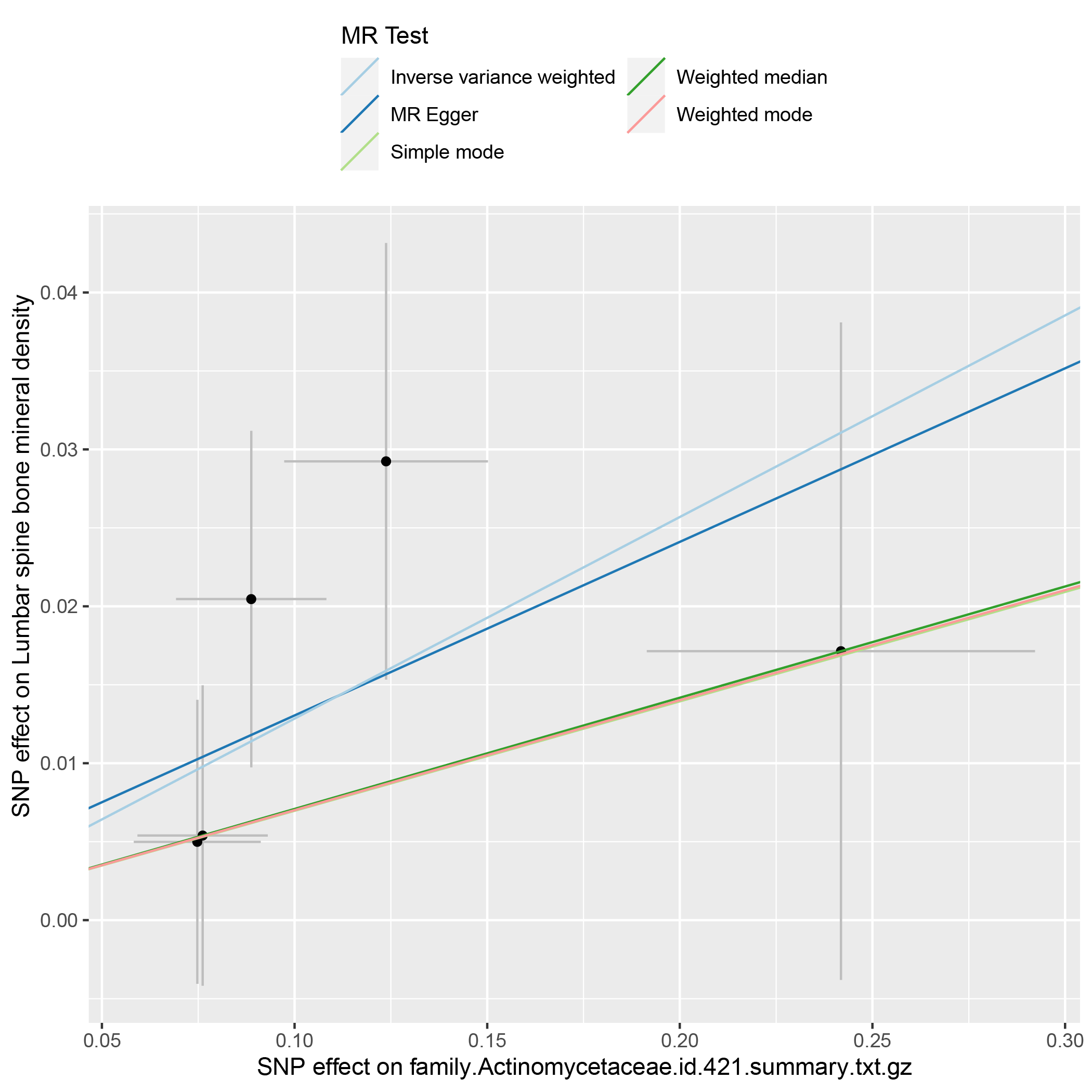


C
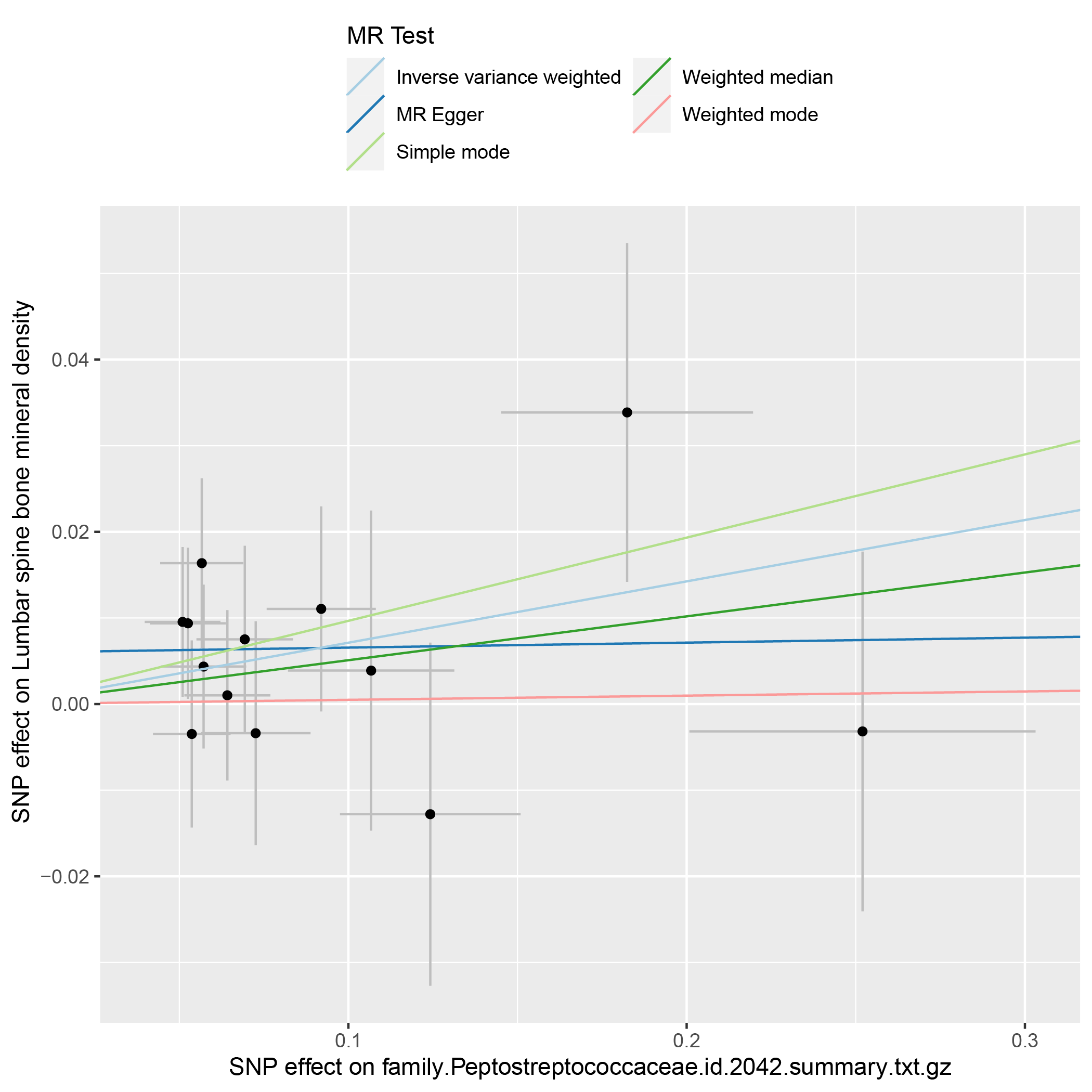
D
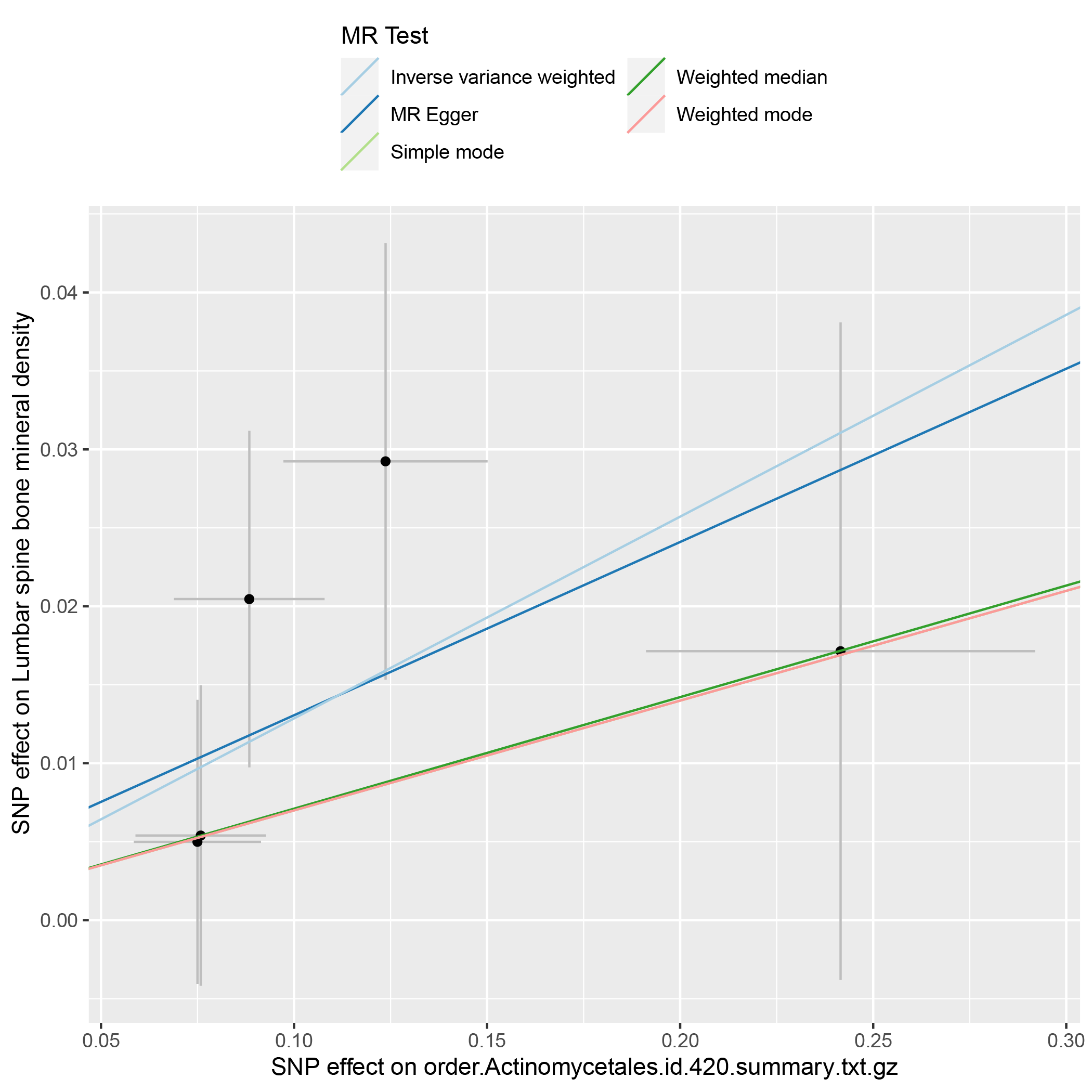


E
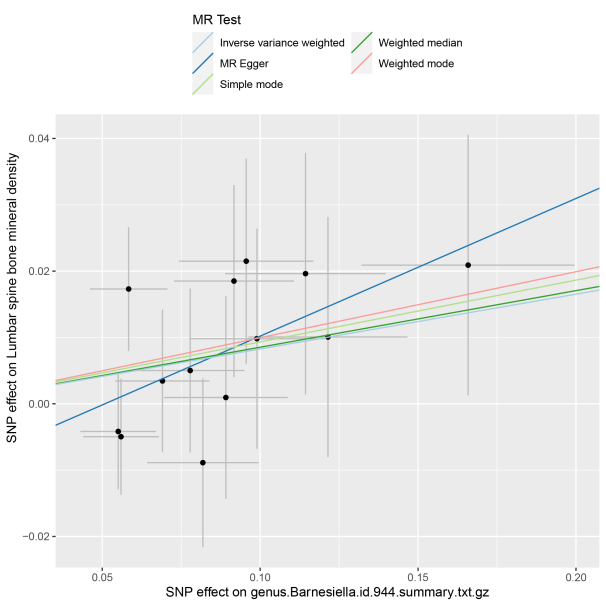
F
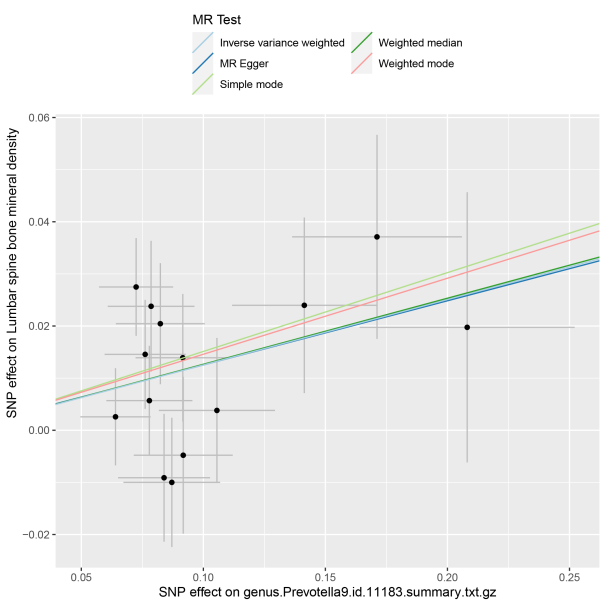
G
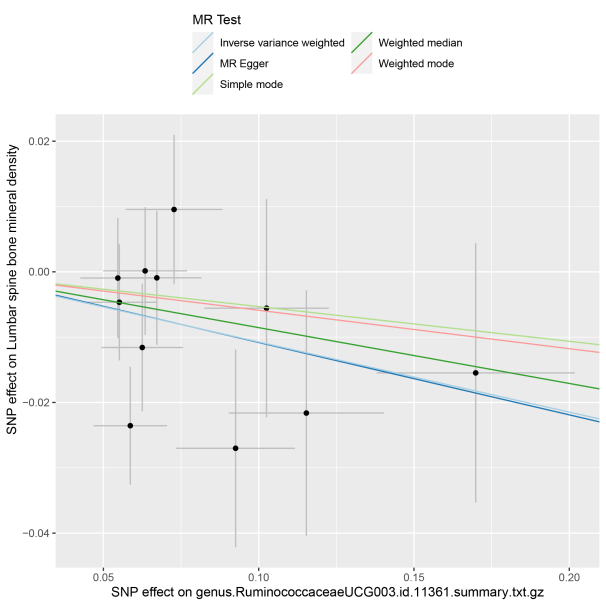
H
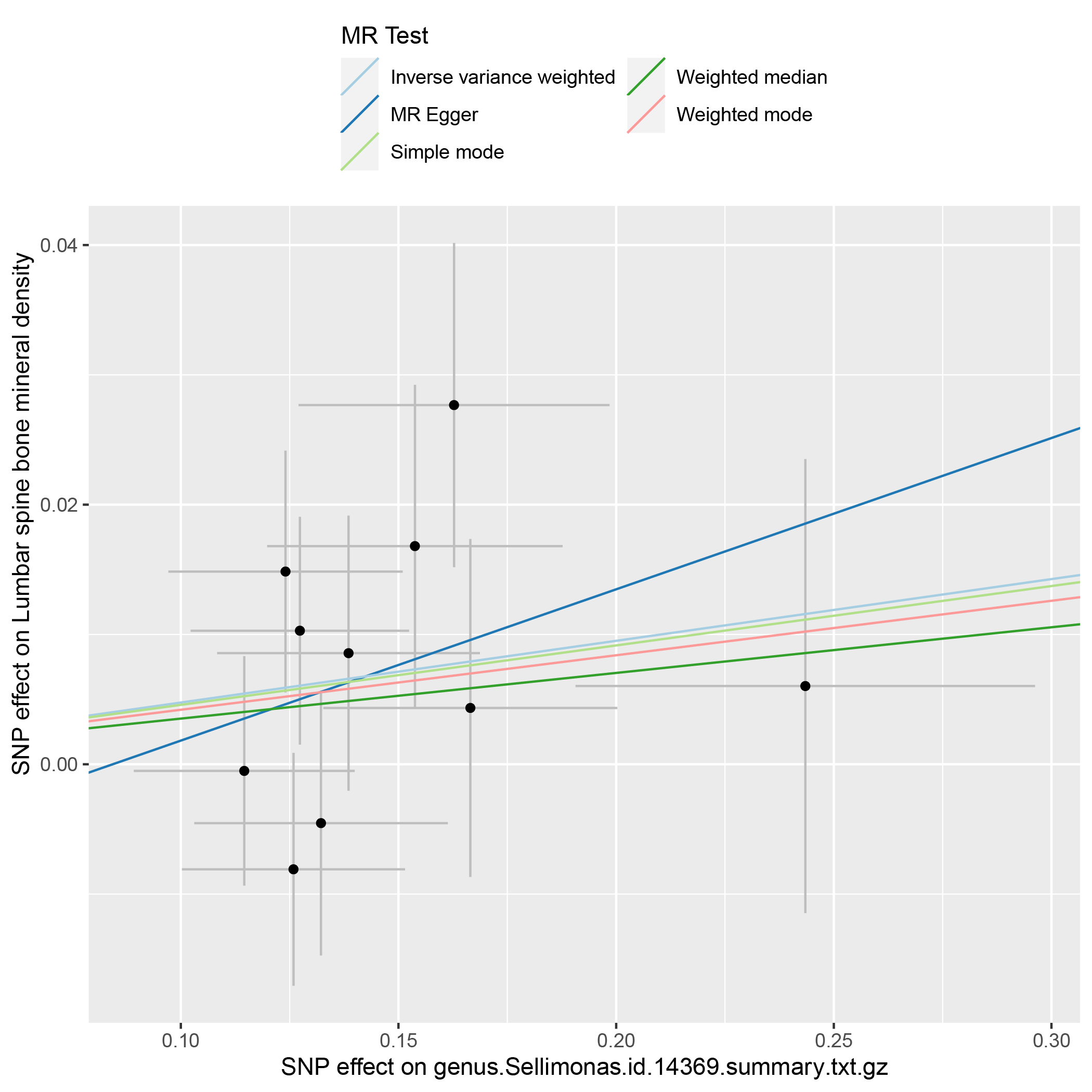


I
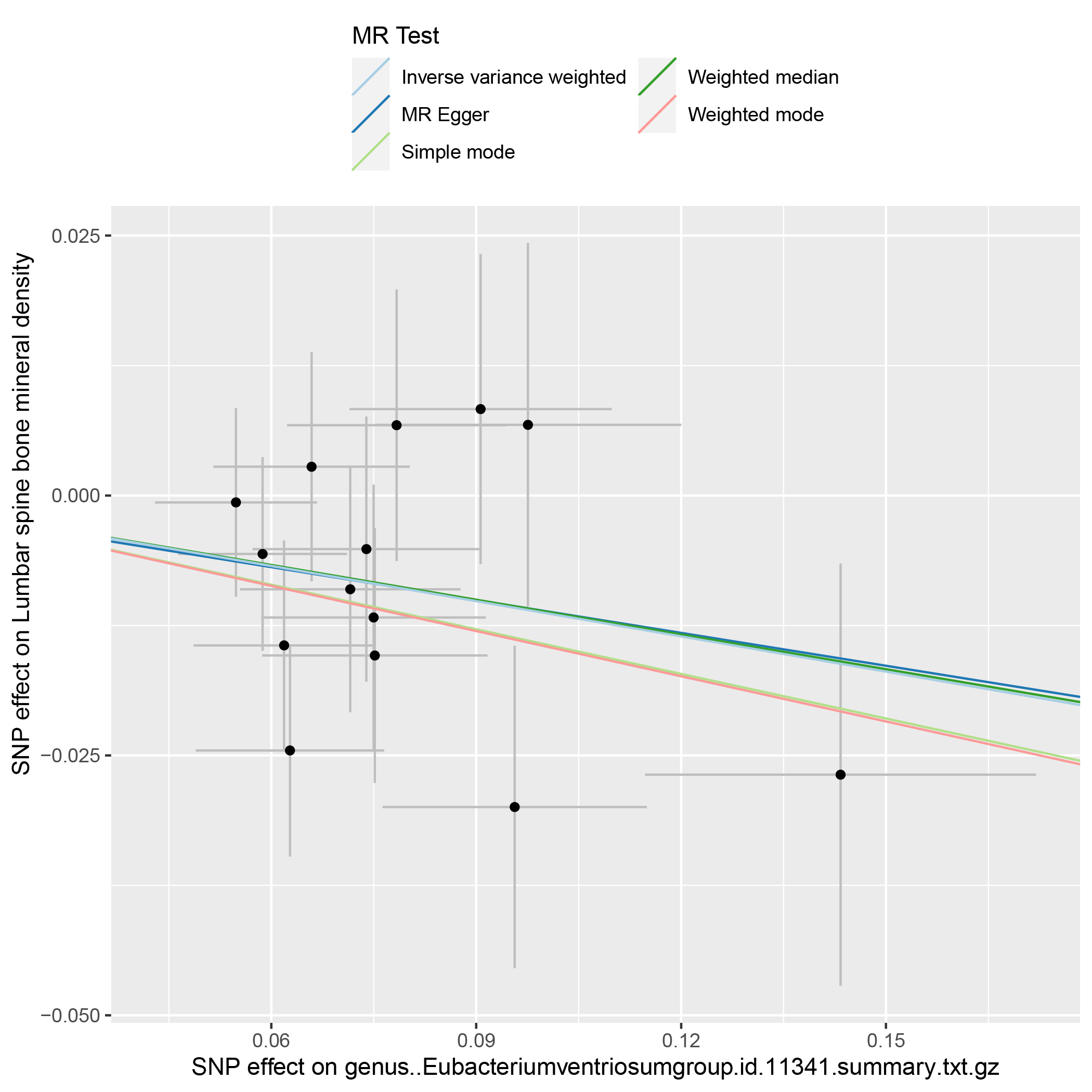


**Supplementary Figure S2.**

Scatter plots of causal estimates of exposure (Specific gut microbiota) on forearm bone mineral density. The slope of each line corresponding to the estimated MR effect in different models, including the conventional IVW, Weighted median, MR-Egger, Simple mode, and Weighted mode. (A): *Family Prevotellaceae*; (B): *Family Rikenellaceae*; (C): *Genus Eubacteriumbrachygroup*; (D): *Genus Coprococcus3*; (E): *Genus LachnospiraceaeUCG001*; (F): *Genus Prevotella9*

A*
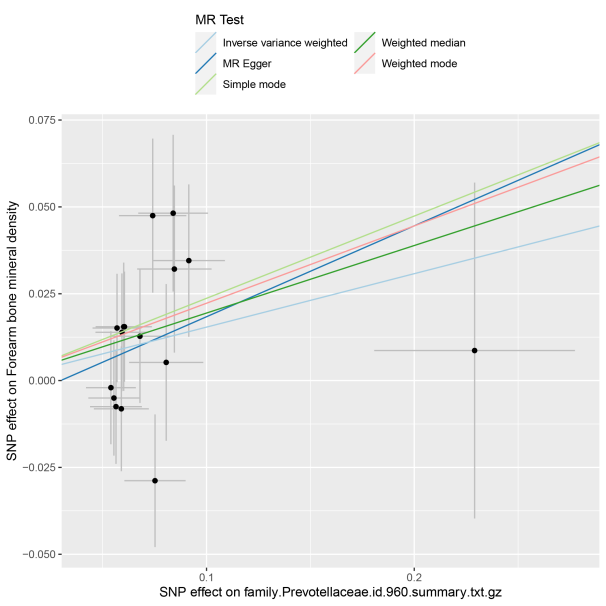
*B*
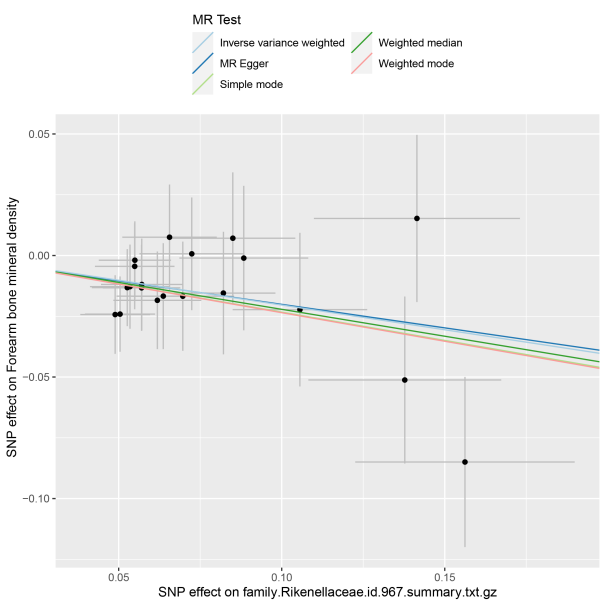
*

C
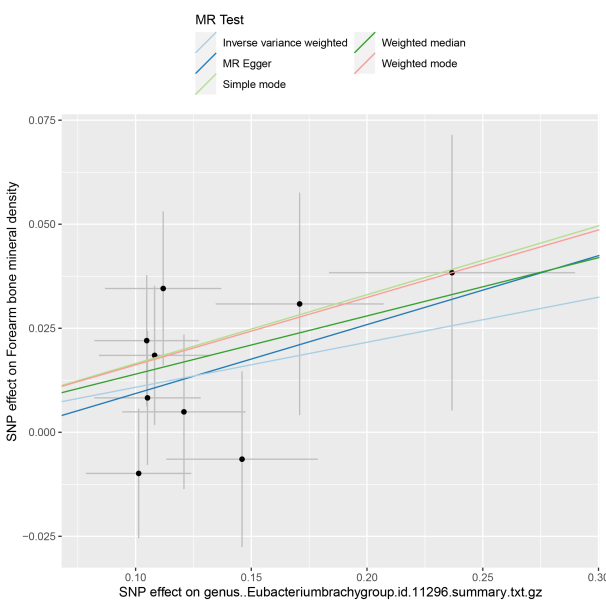
D
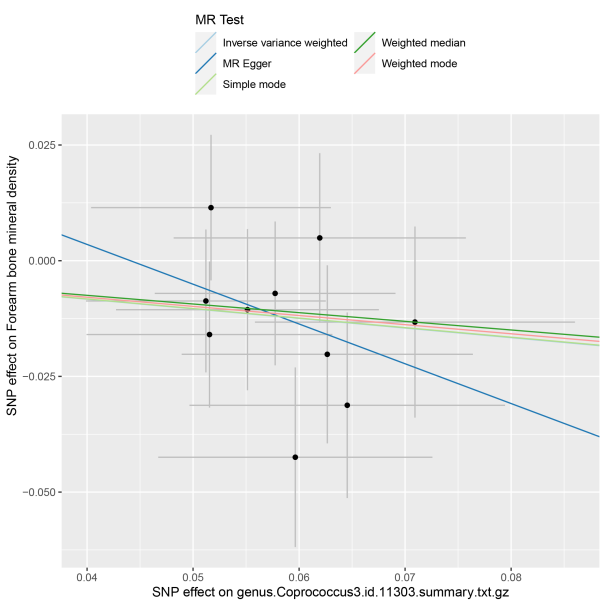


E
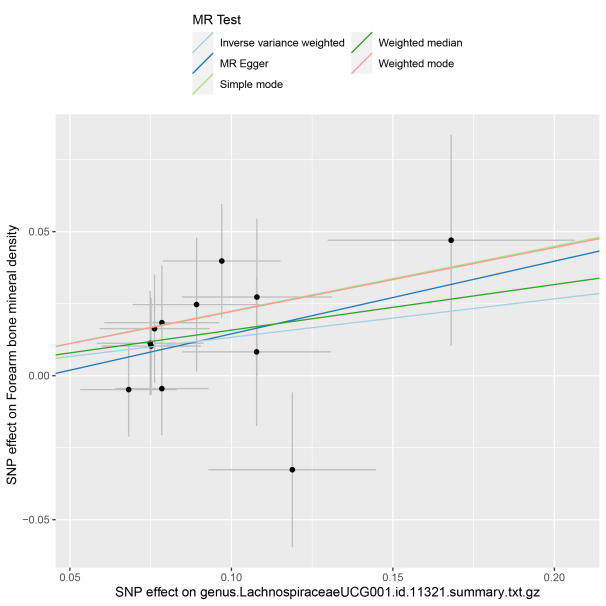
F
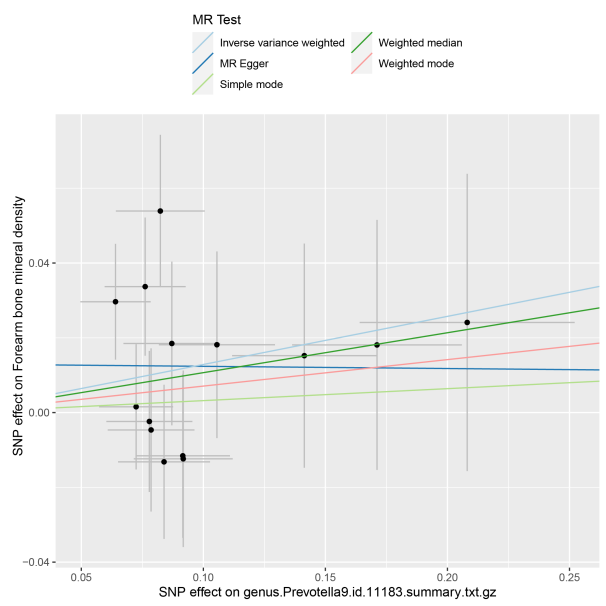


**Supplementary Figure S3.**

Scatter plots of causal estimates of exposure (Specific gut microbiota) on femoral neck bone mineral density. The slope of each line corresponding to the estimated MR effect in different models, including the conventional IVW, Weighted median, MR-Egger, Simple mode, and Weighted mode. (A): *Class Lentisphaeria*; (B): *Family Acidaminococcaceae*; (C): *Family FamilyXIII*; (D): *Family Prevotellaceae*; (E): *Order Victivallales*; (F): *Phylum Lentisphaerae*; (G): *Genus Ruminococcusgauvreauiigroup*; (H): *Genus Olsenella*

A
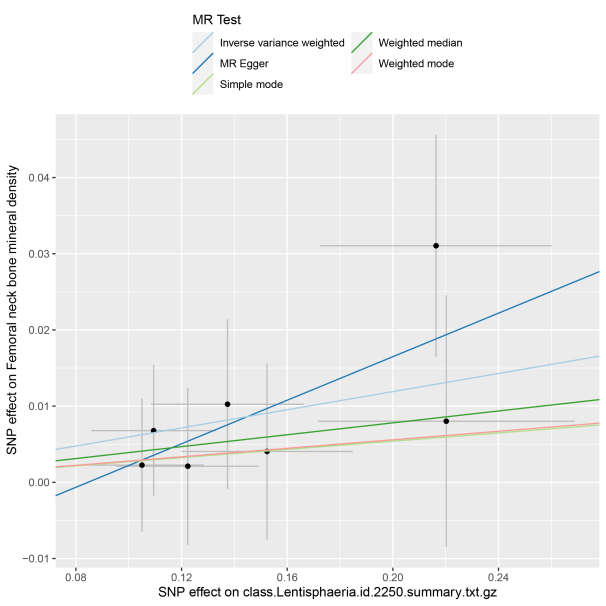
B
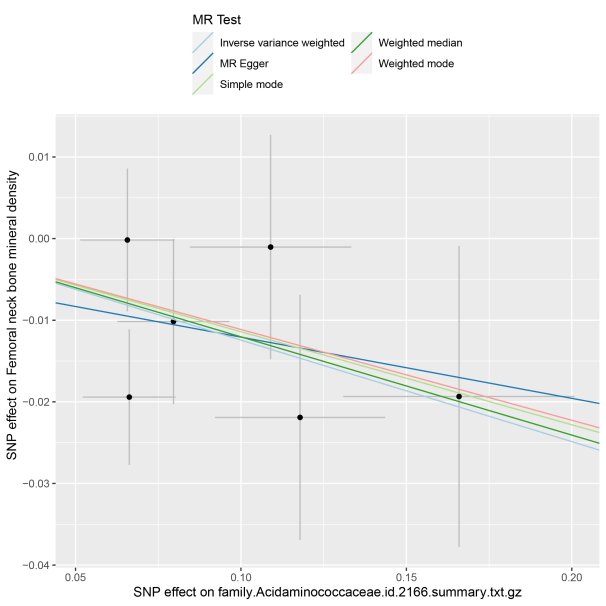


C
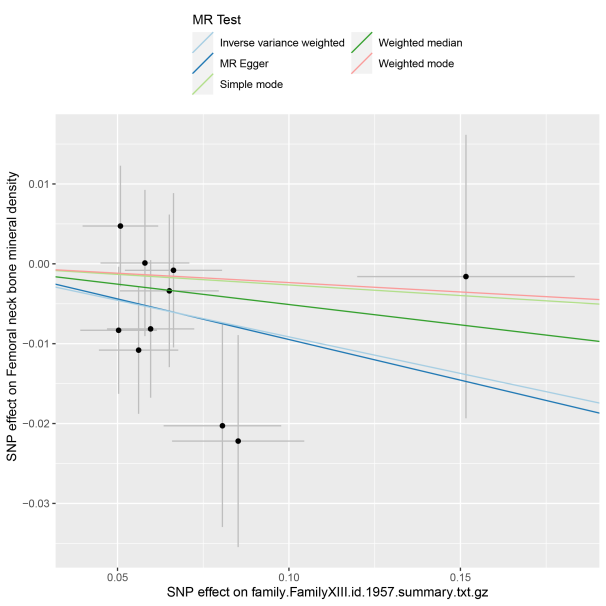
D
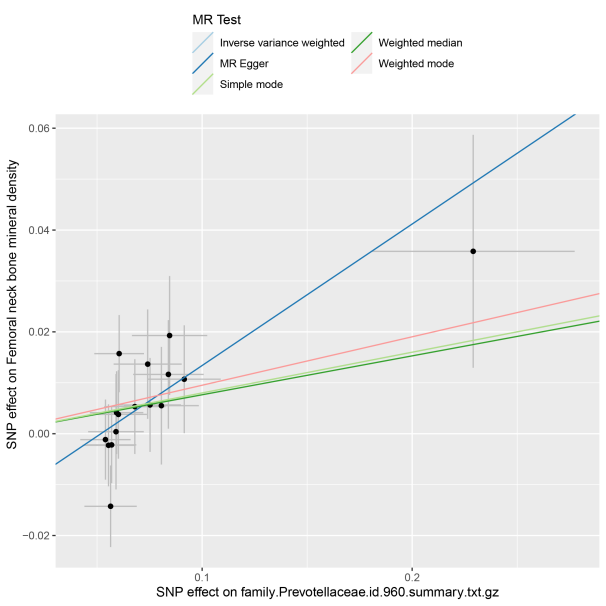


E
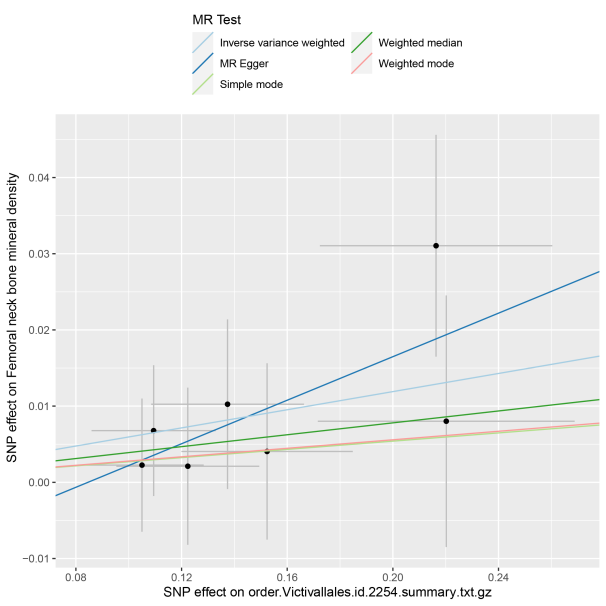
F
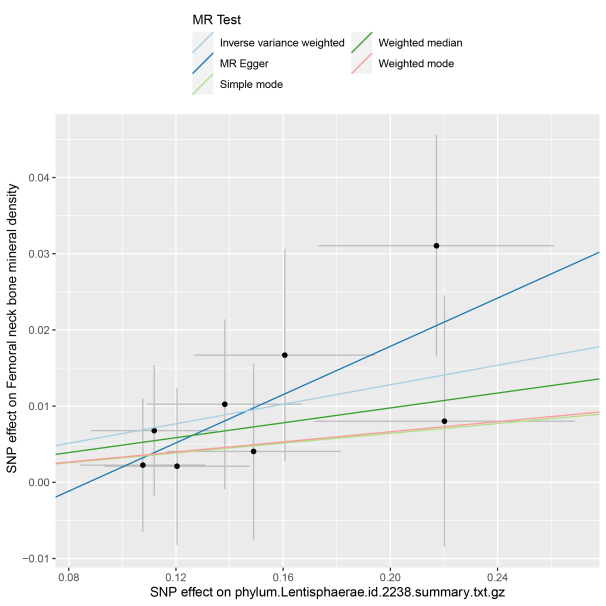


G
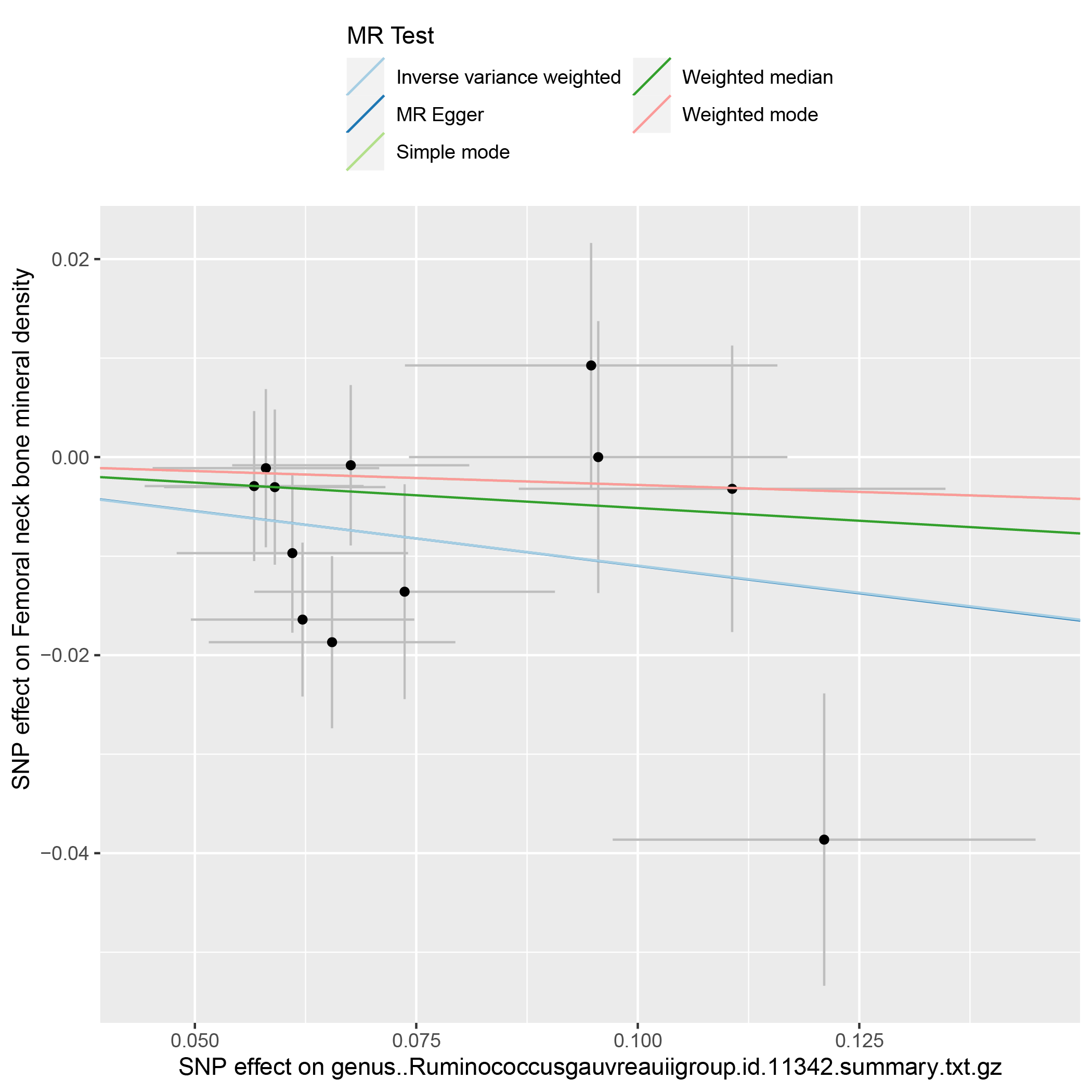
H
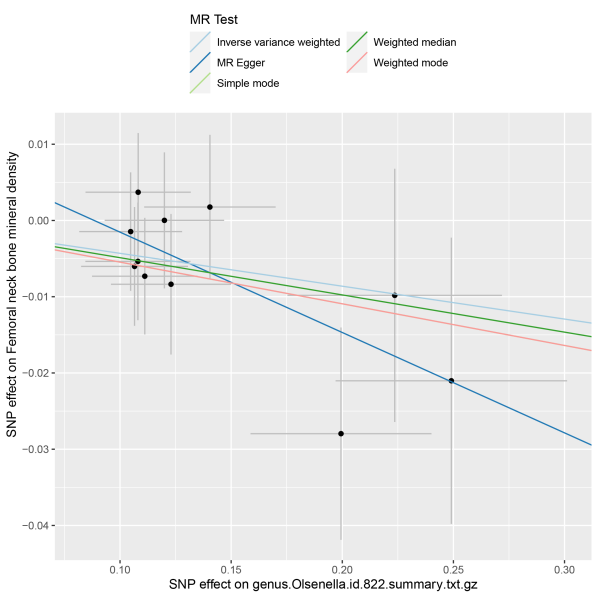


**Supplementary Figure S4.**

Leave-one-out stability tests causal estimates of exposure (Specific gut microbiota) on lumbar spine bone mineral density. Calculate the MR results of the remaining IVs after removing the IVs one by one. (A): *Class Erysipelotrichia*; (B): *Family Actinomycetaceae*; (C): *Family Peptococcaceae*; (D): *Order Actinomycetales*; (E): *Genus Barnesiella*; (F): *Genus Prevotella9*; (G): *Genus RuminococcaceaeUCG003*; (H): *Genus Sellimonas*; (I): *Genus Eubacteriumventriosumgroup*

A
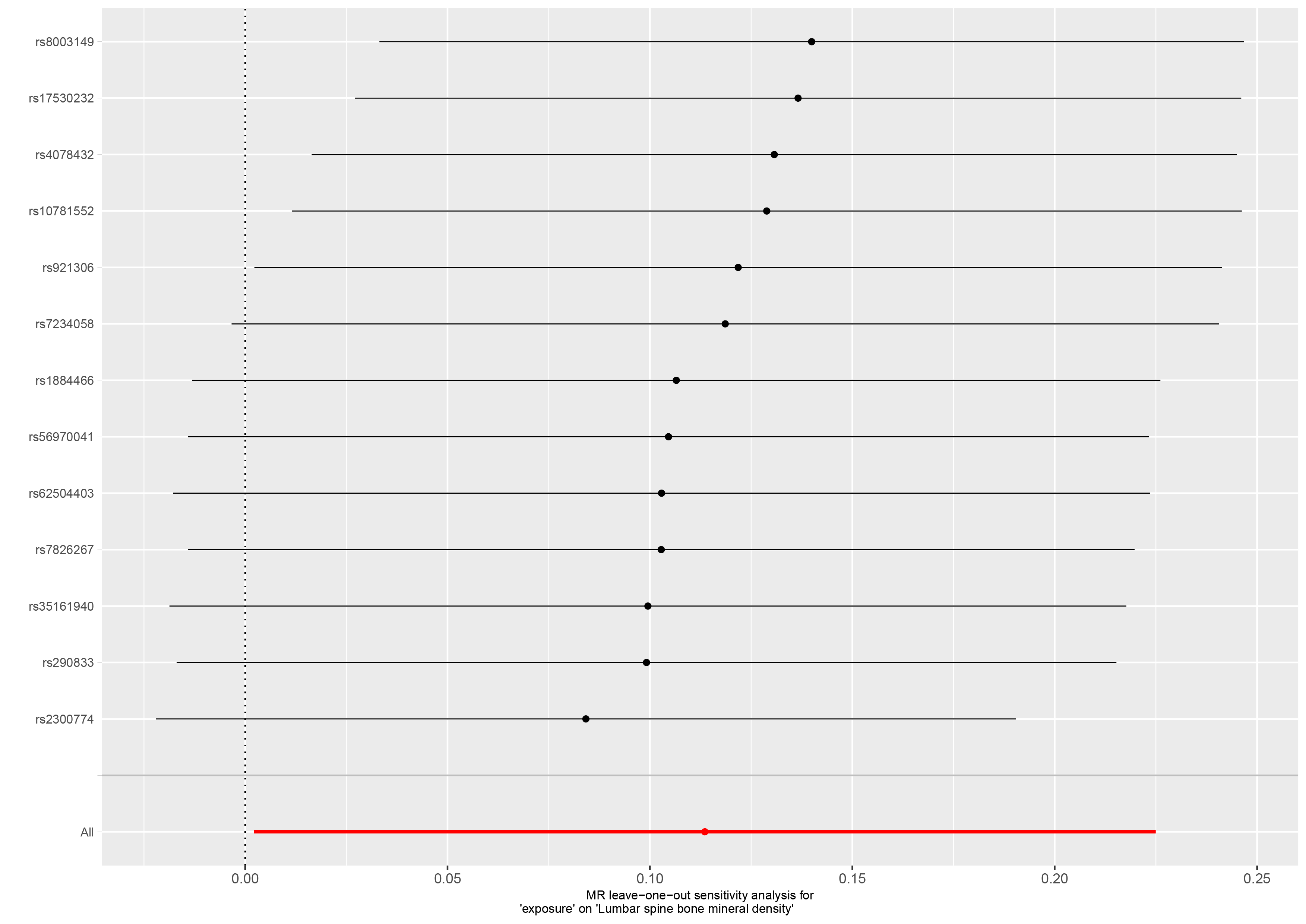
B
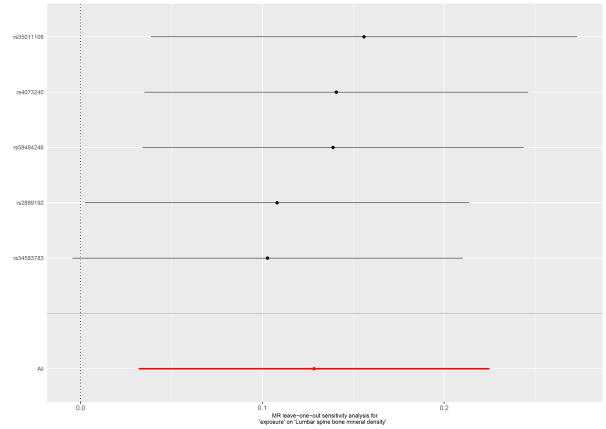
C
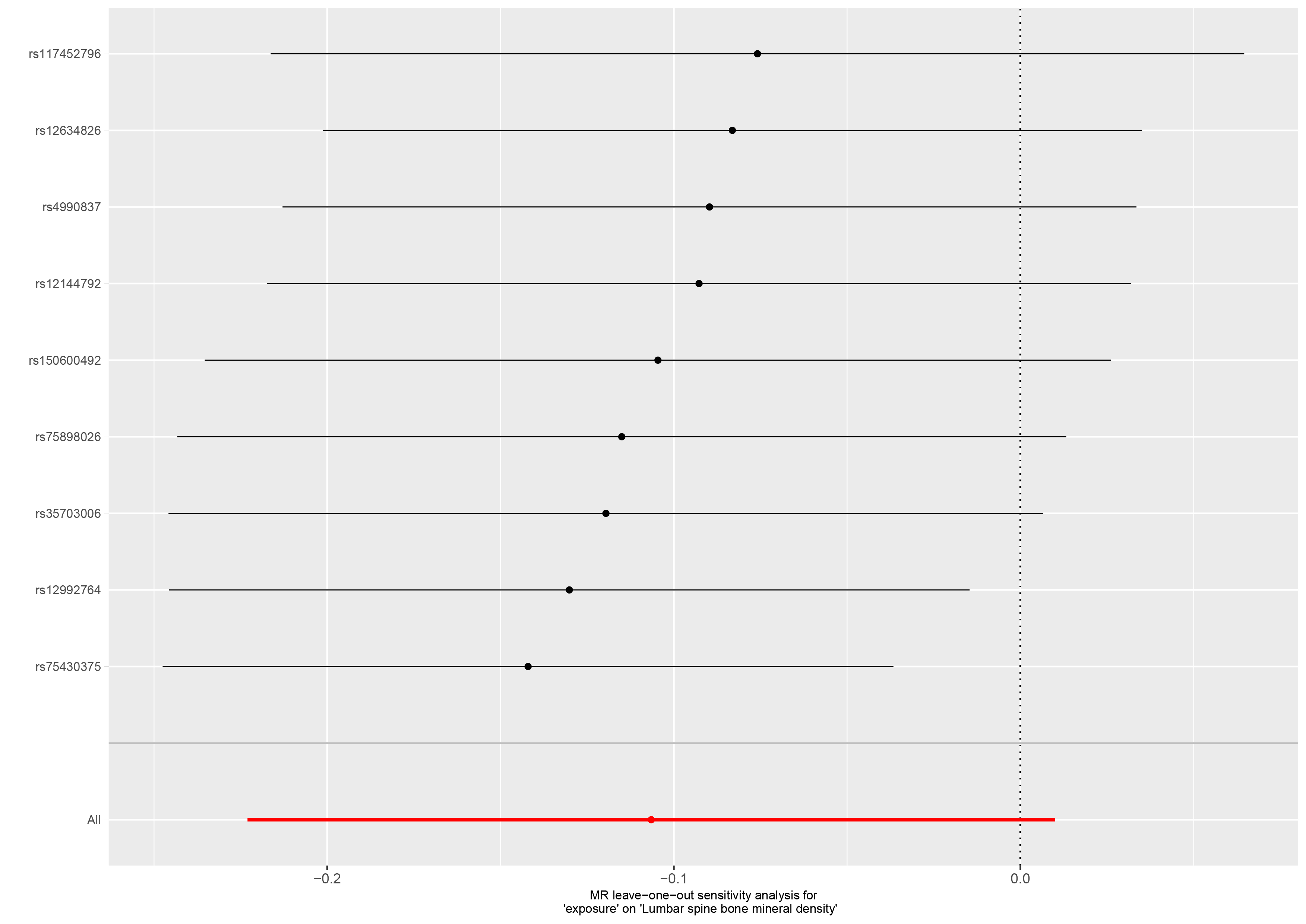
D
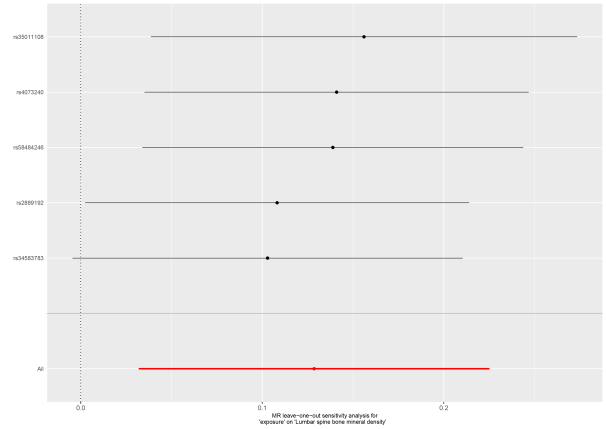


E
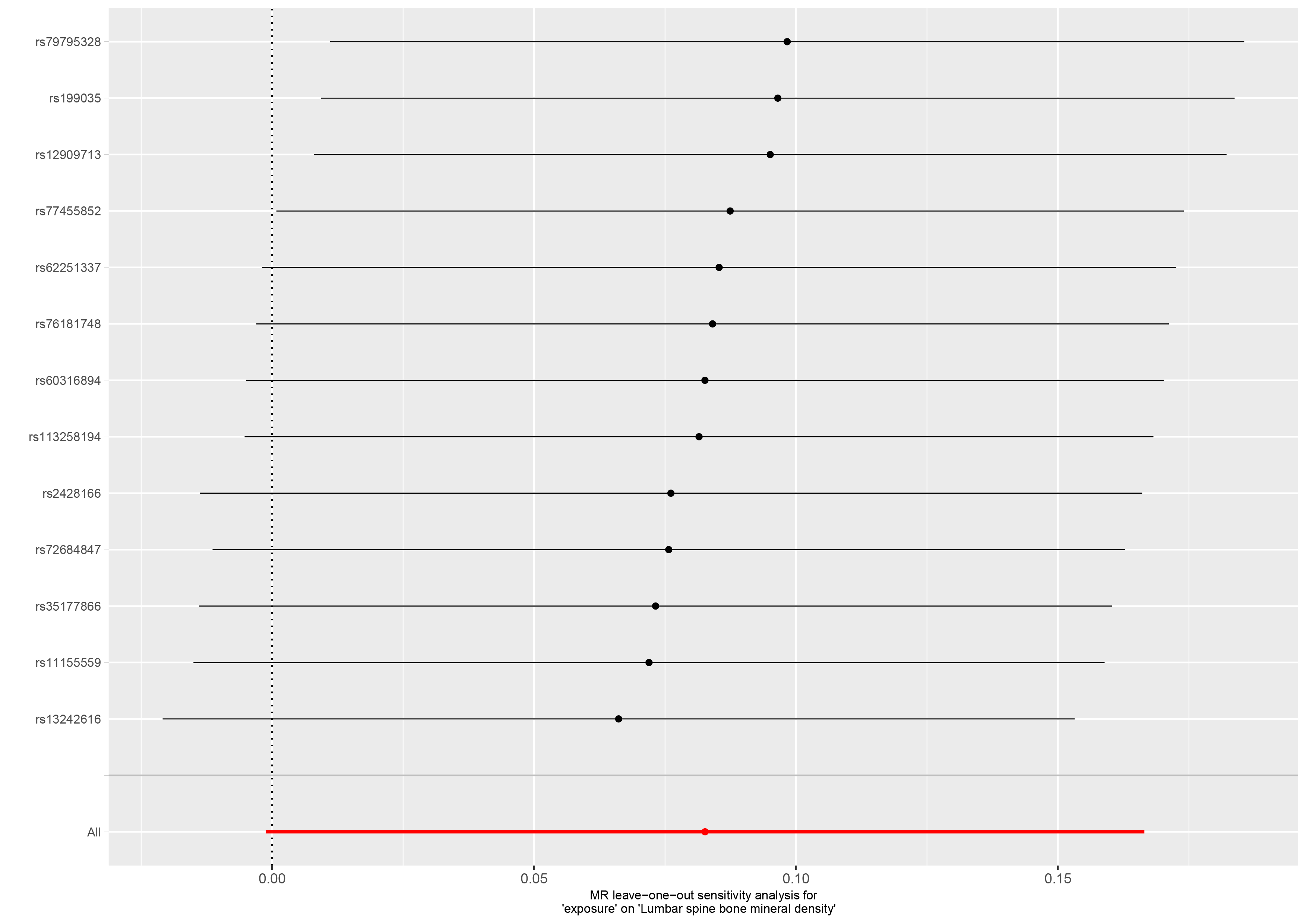
F
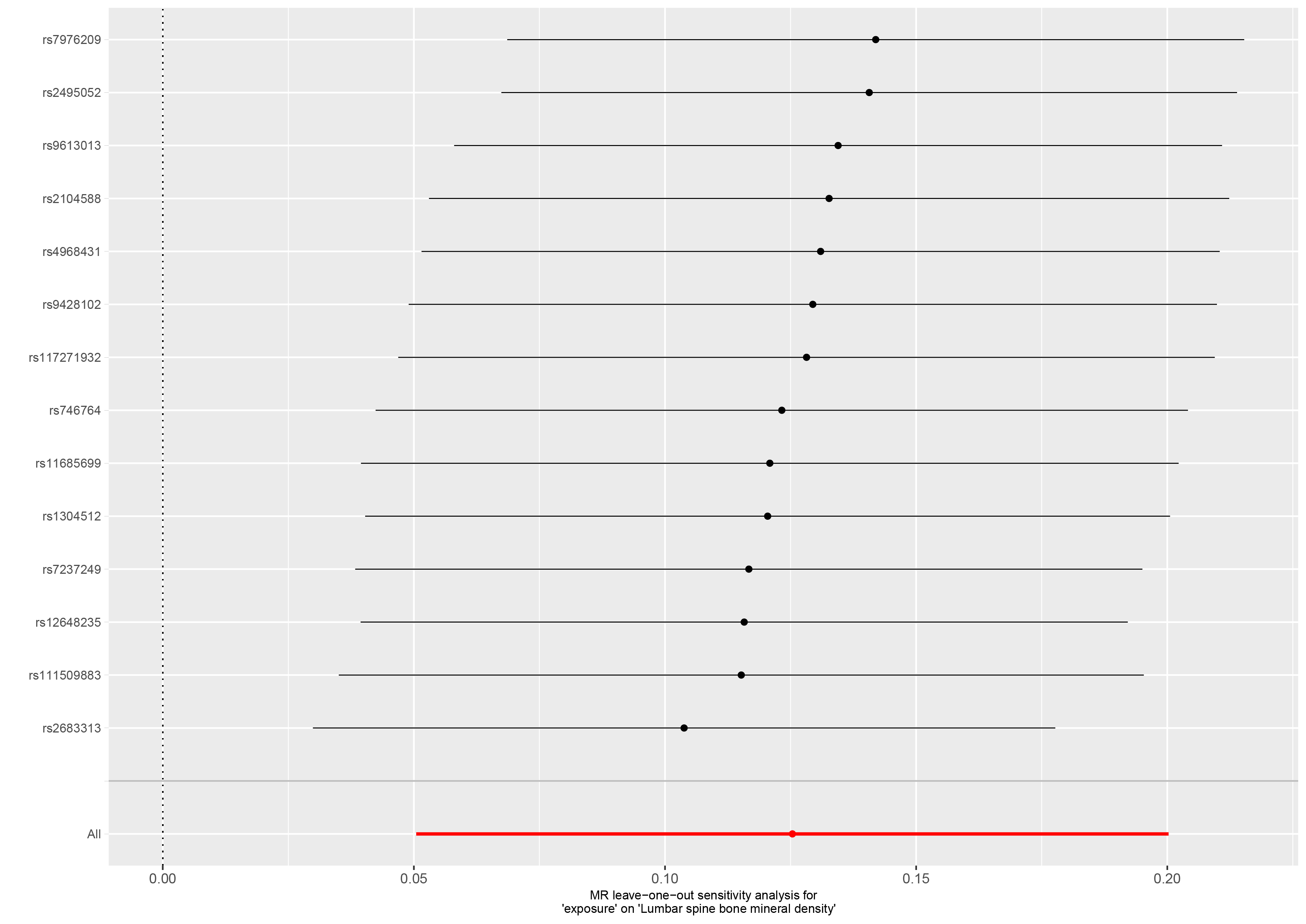


G
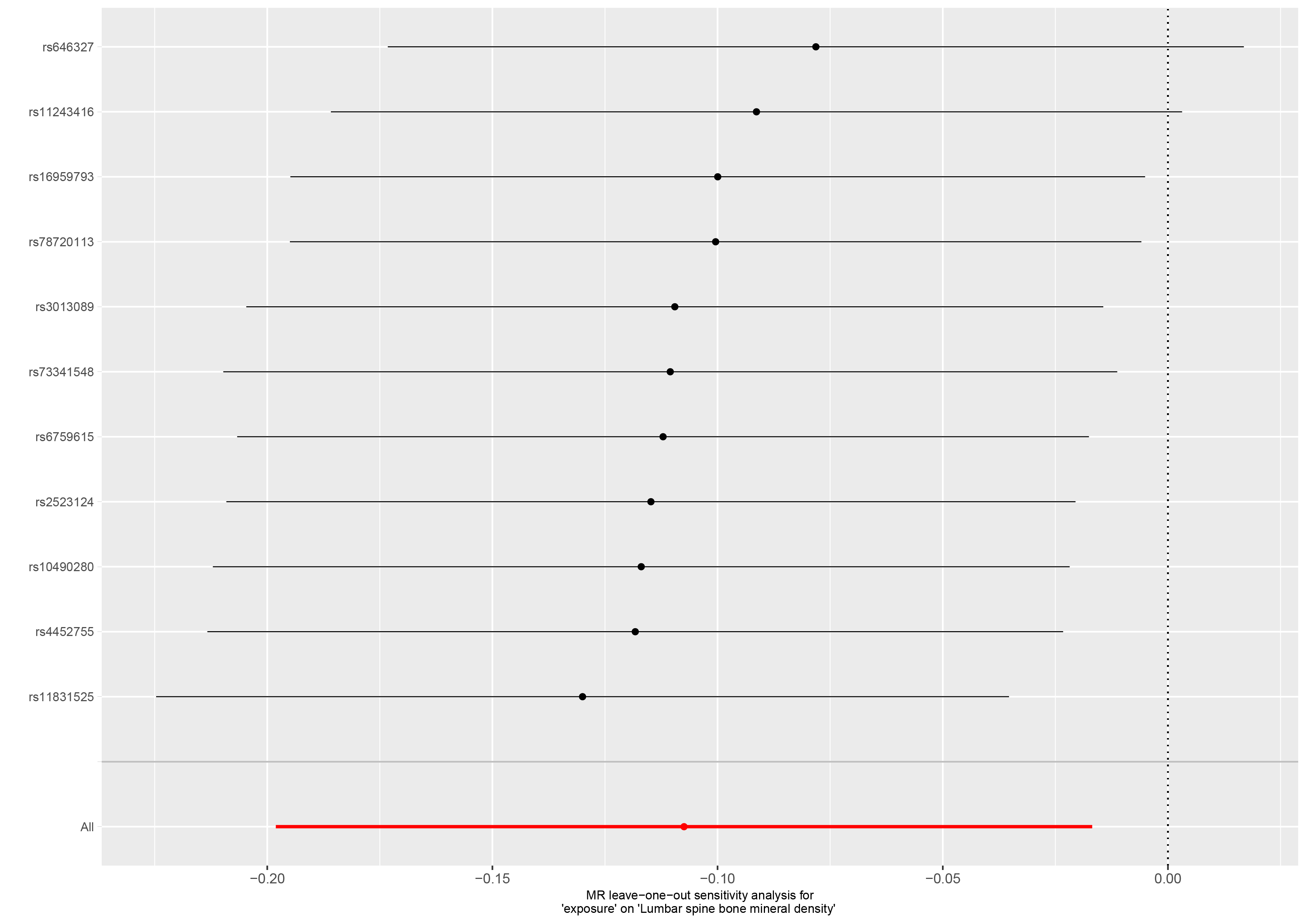
H
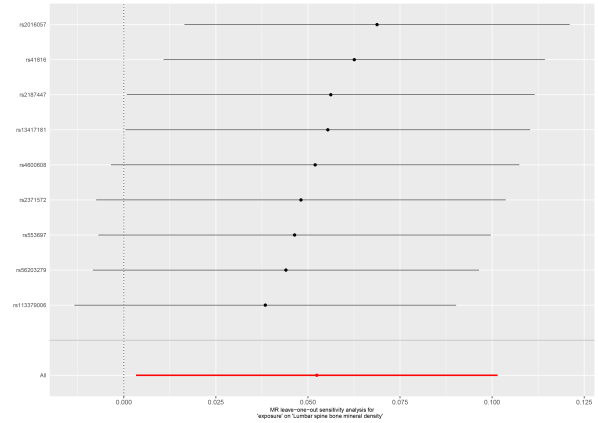


I
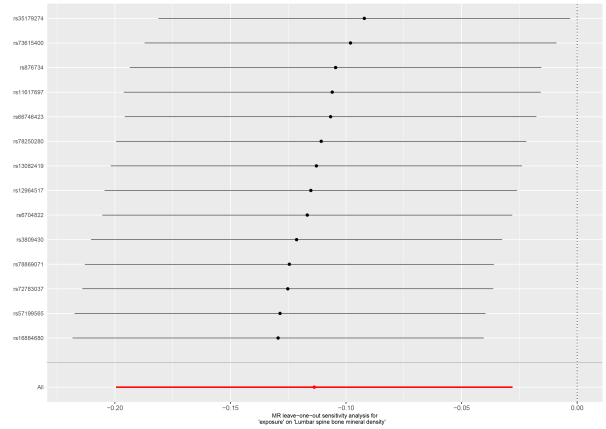


**Supplementary Figure S5.**

Leave-one-out stability tests causal estimates of exposure (Specific gut microbiota) on forearm bone mineral density. Calculate the MR results of the remaining IVs after removing the IVs one by one. (A): *Family Prevotellaceae*; (B): *Family Rikenellaceae*; (C): *Genus Eubacteriumbrachygroup*; (D): *Genus Coprococcus3*; (E): *Genus LachnospiraceaeUCG001*; (F): *Genus Prevotella9*

A*
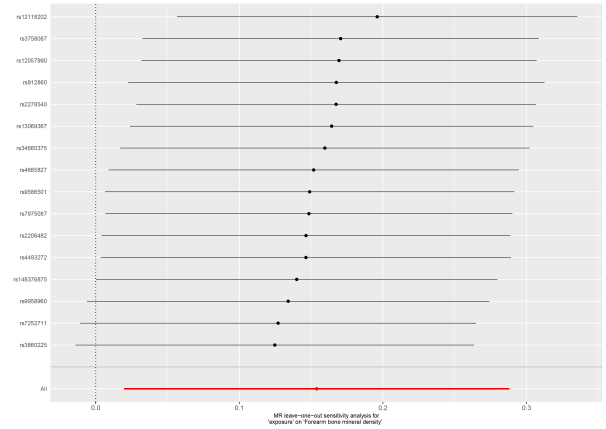
*B*
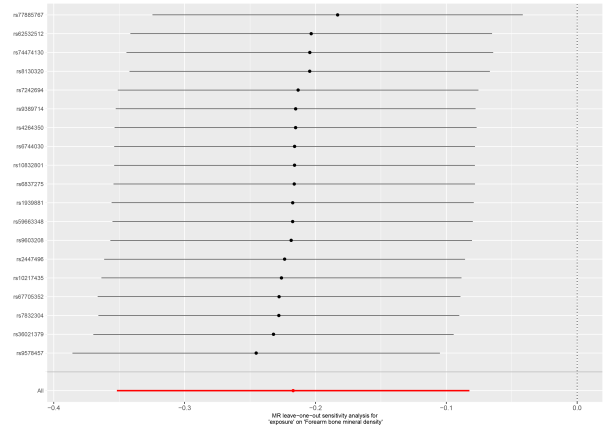
*

C*
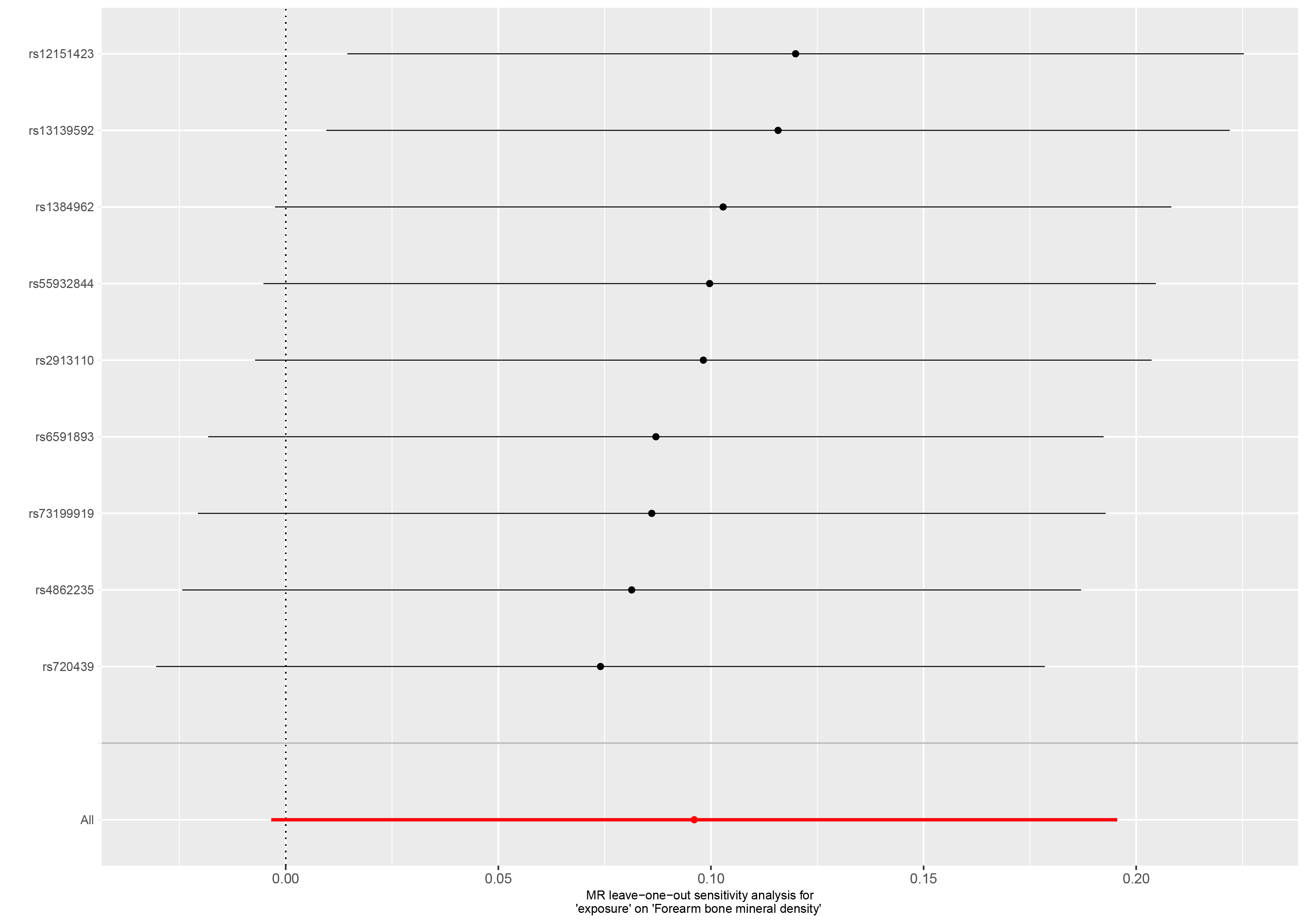
*D*
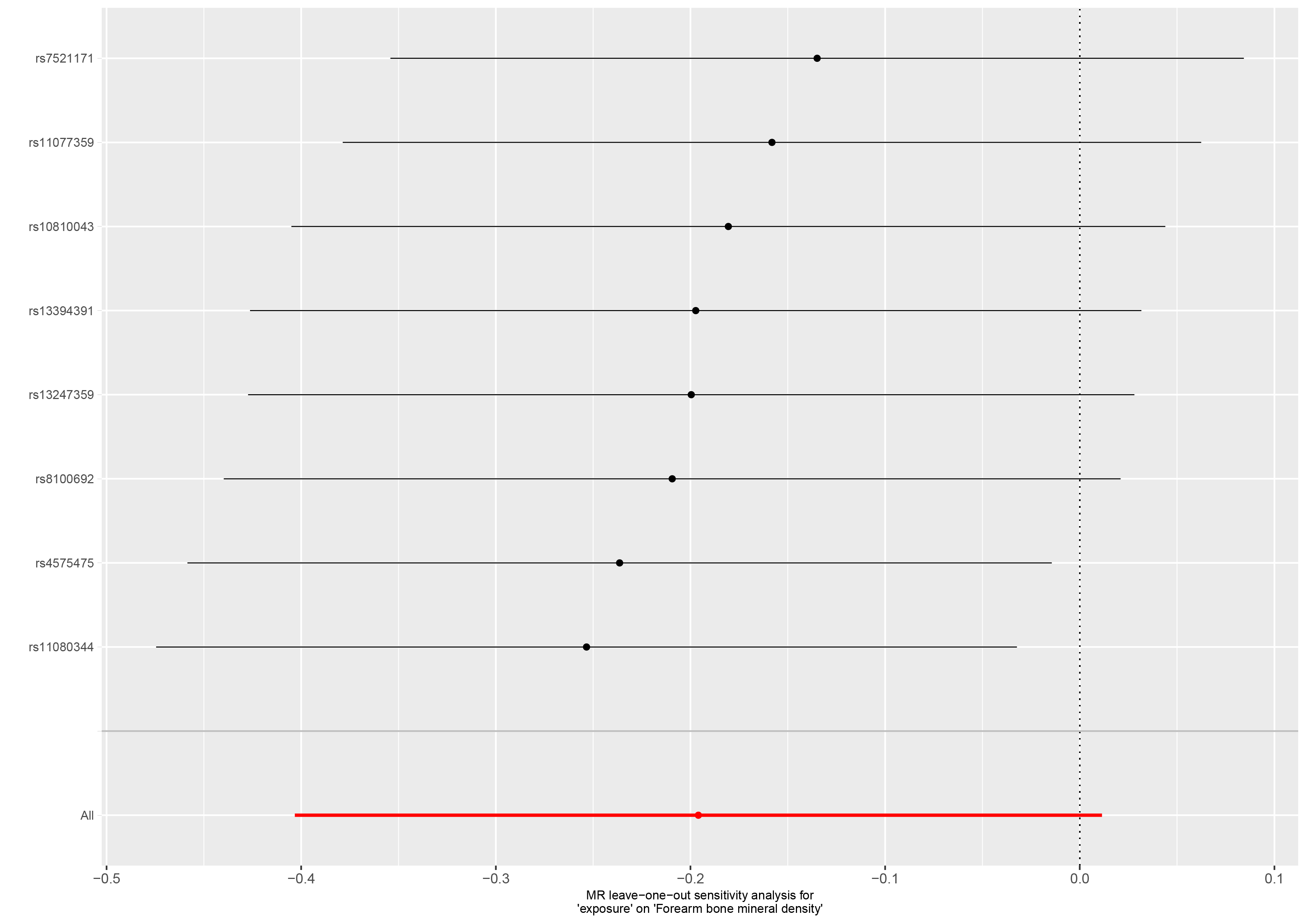
*

E*
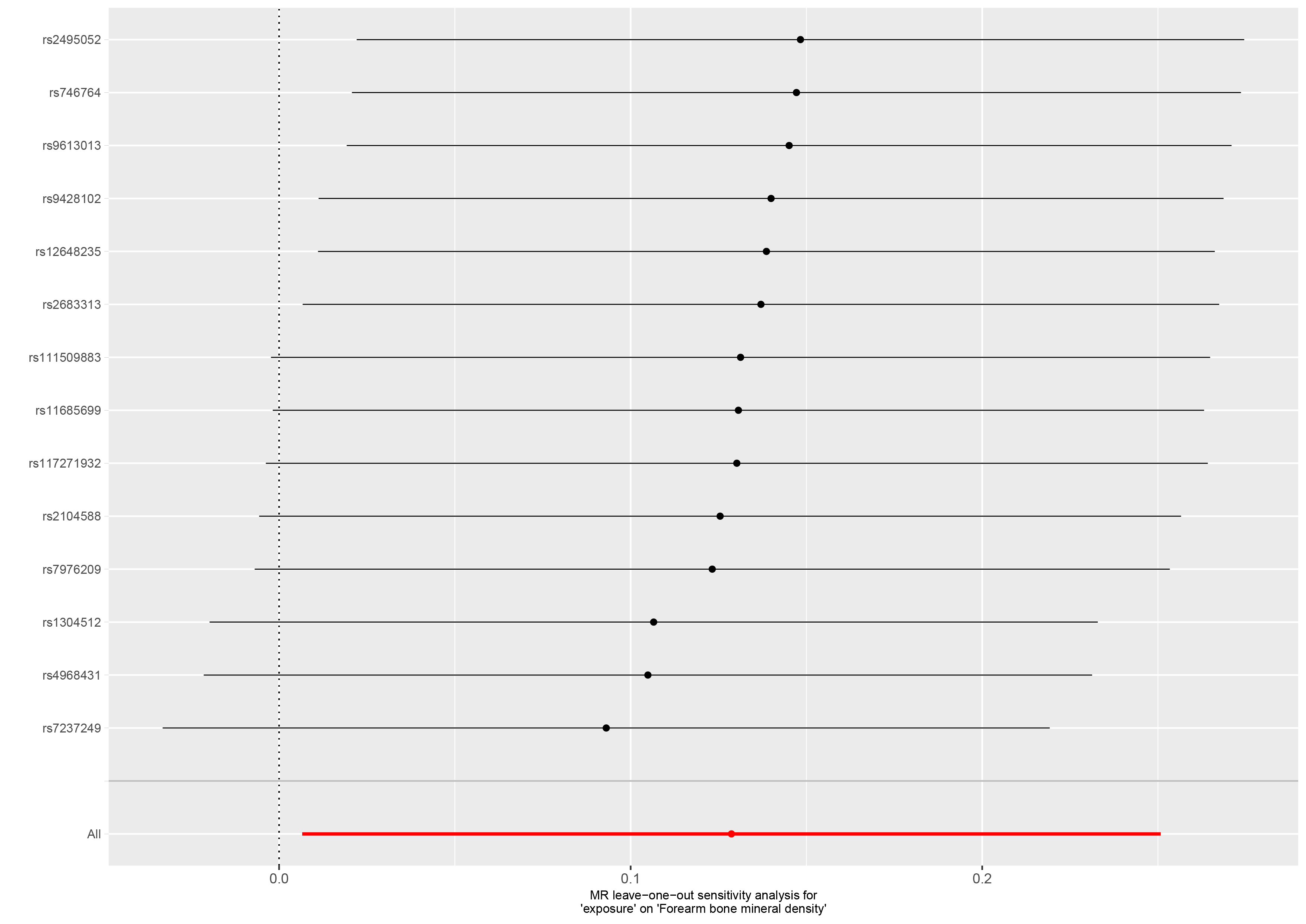
*F*
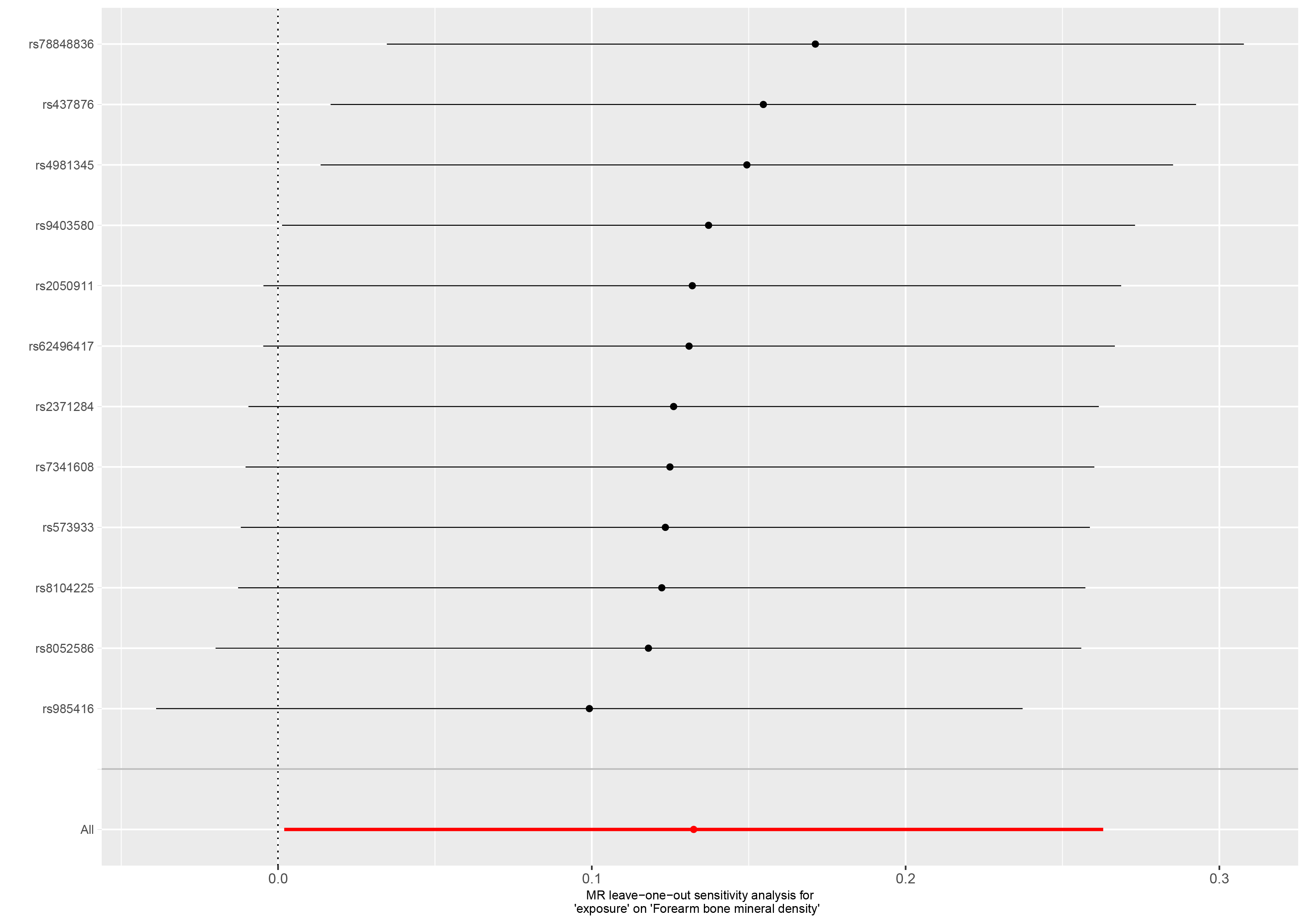
*

**Supplementary Figure S6.**

Leave-one-out stability tests causal estimates of exposure (Specific gut microbiota) on femoral neck bone mineral density. Calculate the MR results of the remaining IVs after removing the IVs one by one. (A): *Class Lentisphaeria*; (B): *Family Acidaminococcaceae*; (C): *Family FamilyXIII*; (D): *Family Prevotellaceae*; (E): *Order Victivallales*; (F): *Phylum Lentisphaerae*; (G): *Genus Ruminococcusgauvreauiigroup*; (H): *Genus Olsenella*

A
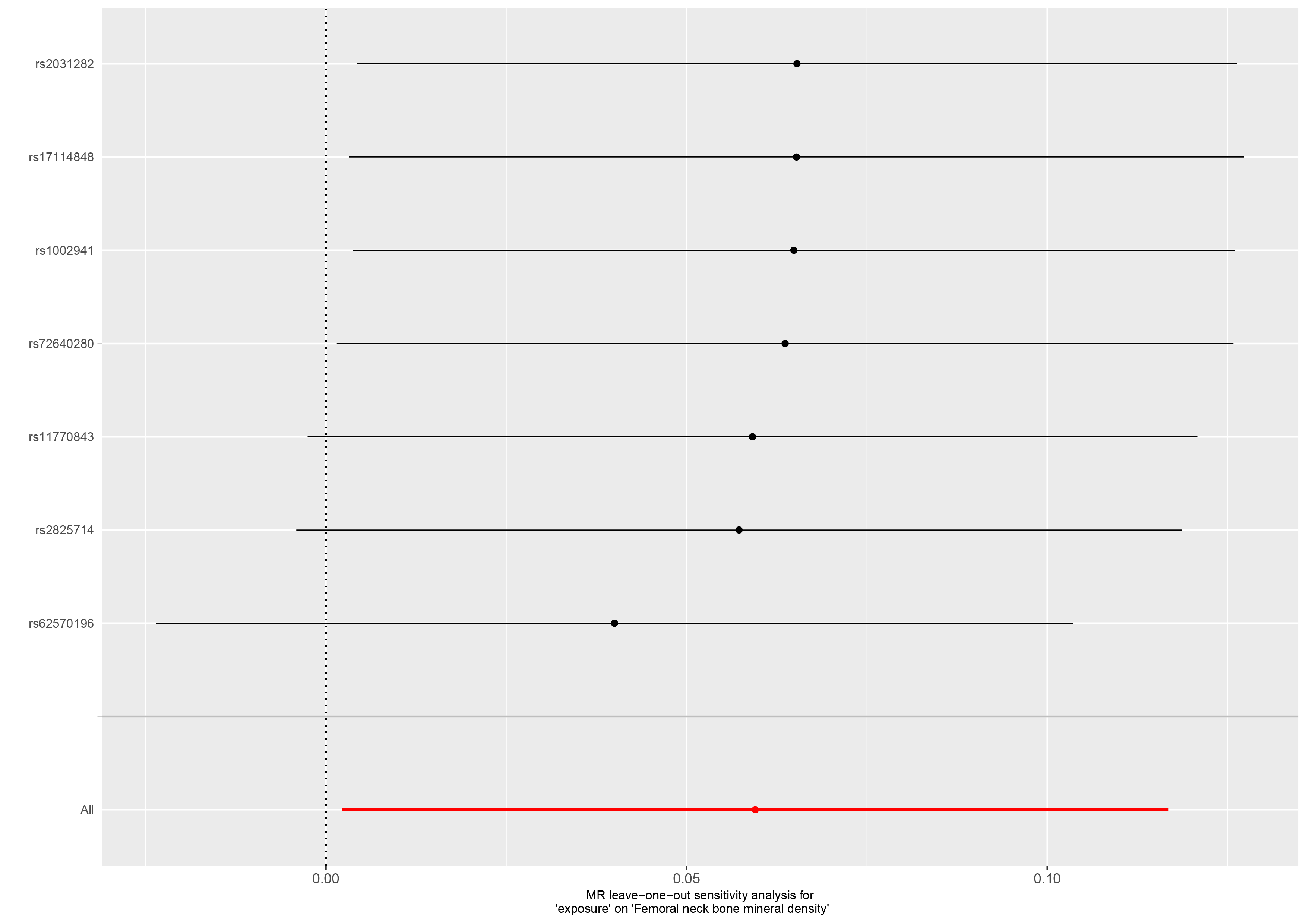
B
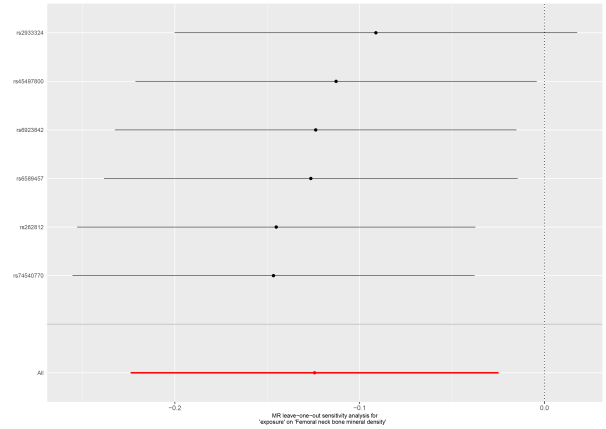


C
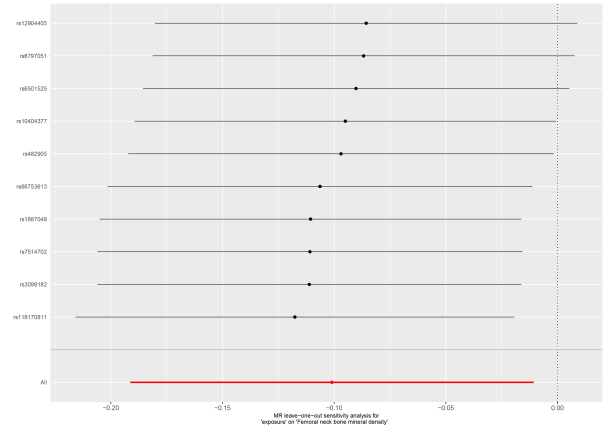
D
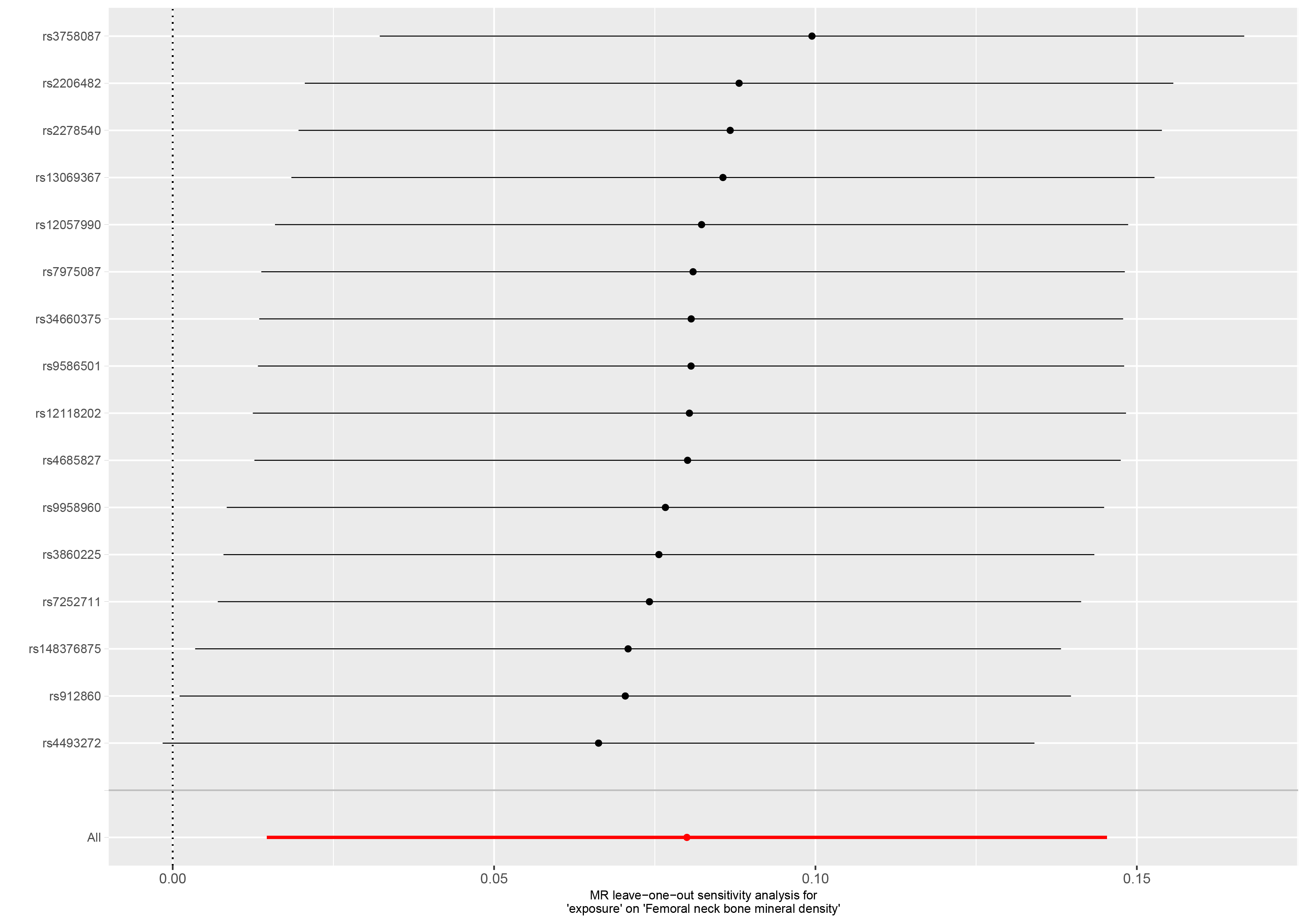


E
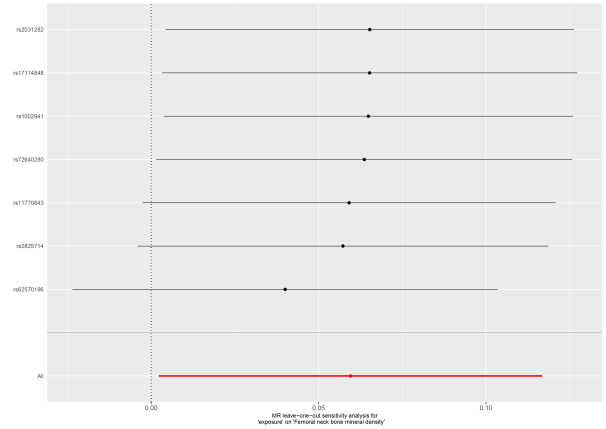
F
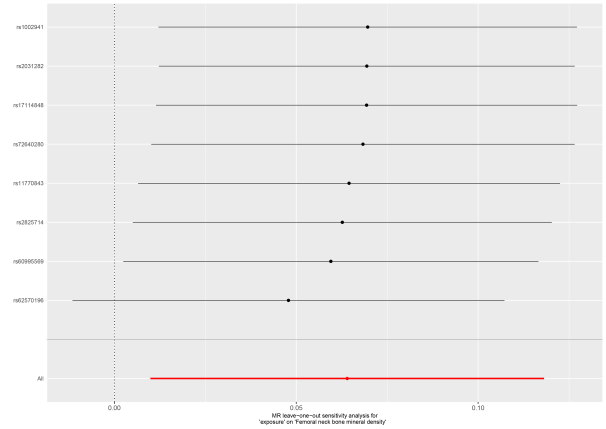


G
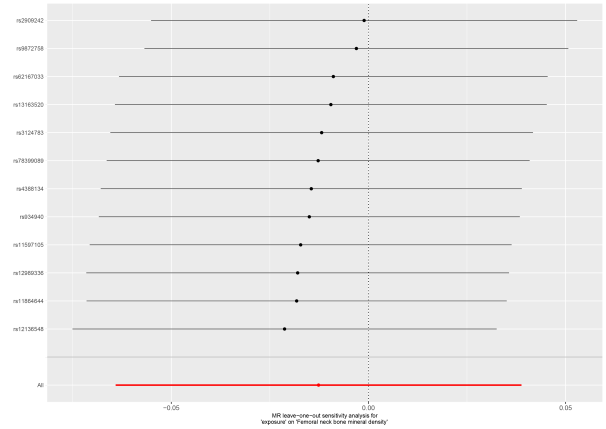
H
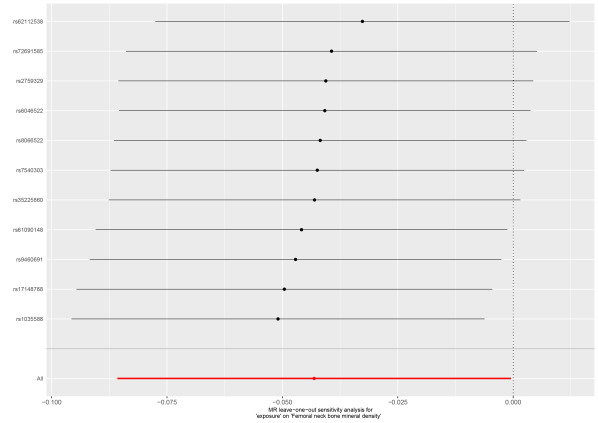

Supplement: Supplementary file 1 [file Table_1.docx]
